# Dissection of Superior Alleles for Yield-Related Traits and Their Distribution in Important Cultivars of Wheat by Association Mapping

**DOI:** 10.3389/fpls.2020.00175

**Published:** 2020-03-02

**Authors:** Xiaojun Li, Xin Xu, Weihua Liu, Xiuquan Li, Xinming Yang, Zhengang Ru, Lihui Li

**Affiliations:** ^1^ School of Life Science and Technology, Henan Institute of Science and Technology, Collaborative Innovation Center of Modern Biological Breeding, Henan Province, Henan Provincial Key Laboratory of Hybrid Wheat, Xinxiang, China; ^2^ Department of Life Sciences and Technology, Xinxiang University, Xinxiang, China; ^3^ The National Key Facility for Crop Gene Resources and Genetic Improvement (NFCRI), Institute of Crop Sciences, Chinese Academy of Agricultural Sciences, Beijing, China

**Keywords:** bread wheat, association mapping, superior allele, yield-related traits, founder parent, widely grown cultivar

## Abstract

Uncovering the genetic basis of yield-related traits is important for molecular improvement of wheat cultivars. In this study, a genome-wide association study was conducted using the wheat 55K genotyping assay and a diverse panel of 384 wheat genotypes. The accessions used included 18 founder parents and 15 widely grown cultivars with annual maximum acreages of over 667,000 ha, and the remaining materials were elite cultivars and breeding lines from several major wheat ecological areas of China. Field trials were conducted in five major wheat ecological regions of China over three consecutive years. A total of 460 significant loci were detected for eight yield-related traits. Forty-five superior alleles distributed over 31 loci for which differences in phenotypic values grouped by single nucleotide polymorphism (SNP) reached significant levels (P < 0.05) in nine or more environments, were detected; some of these loci were previously reported. Eleven of the 31 superior allele loci on chromosomes 4A, 5A, 3B, 5B, 6B, 7B, 5D, and 7D had pleiotropic effects. For example, AX-95152512 on 5D was simultaneously related to increased grain weight per spike (GWS) and decreased plant height (PH); AX-109860828 on 5B simultaneously led to a high 1,000-kernel weight (TKW) and short PH; and AX-111600193 on 4A was simultaneously linked to a high TKW and GWS, and short PH. The favorable alleles in each accession ranged from 2 to 30 with an average of 16 at the thirty-one loci in the population, and six accessions (Zhengzhou683, Suzhou7829, Longchun7, Ningmai6, Yunmai35 and Zhen7630) contained more than 27 favorable alleles. A significant association between the number of favorable alleles and yield was observed (r = 0.799, p < 0.0001), suggesting that pyramiding multiple QTL with marker-assisted selection may effectively increase yield of wheat. Furthermore, distribution of superior alleles in founder parents and widely grown cultivars was also discussed here. This study is useful for marker-assisted selection for yield improvement and dissecting the genetic mechanism of important cultivars in wheat.

## Introduction

Bread wheat (*Triticum aestivum* L.) is one of the most important staple crops globally. In the last decade, wheat yield and total production have increased signiﬁcantly due to the release and utilization of new cultivars worldwide, however the current yield gain trends are still insufﬁcient to feed a global population of 9 billion by 2050 (Ray et al., 2012). In particular, some authors (Reif et al., 2005; Tian et al., 2005) have suggested that the genetic diversity of wheat has been increasingly narrowed; thus, it is very important to discover more genetic loci controlling yield-related traits to broaden the genetic variation and accelerate varietal improvement in future wheat breeding.

Most important agronomic traits in plants are controlled by multiple genes and are significantly influenced by the environment. The method most widely used way to identify quantitative trait loci (QTLs) in plants generally depends on bi-parental population-based linkage analysis. To date, many QTLs in wheat such as those associated with yield-related traits (Cui et al., 2014), quality (Cabrera et al., 2015), preharvest sprouting tolerance (Kulwal et al., 2005) and ﬂag leaf traits (Wu Q. et al., 2015) have been identified using linkage mapping studies. However, genes identified by this method are restricted to one bi-parental experimental population, and the limited number of reorganization events occurring at genetic loci during the development of the mapping population leads to QTLs with low resolution (Holland, 2007). Common QTLs across different mapping populations are also rarely defined or explored (Cui et al., 2014). Therefore, there is limited potential for these detected QTLs to be used in practical plant breeding.

Alternatively, association mapping is a population-based survey method used to identify trait-marker relationships based on linkage disequilibrium (LD). Compared with traditional linkage analysis, association studies of natural diversity panels can provide higher resolution in terms of defining the genomic position of a gene or QTL. Early association studies in plants used the candidate gene approach to identify specific single nucleotide polymorphisms (SNPs) or genes controlling traits of interest, such as the *Glu-B1-1* and *Glu1Bx* genes in wheat (Ravel et al., 2006), *dwarf8* gene in maize (*Zea mays* L.) (Thornsberry et al., 2001), flowering time genes in barley (*Hordeumvulgare* L.) (Stracke et al., 2009) and *Arabidopsis* (Aranzana et al., 2005), and genes governing seed oil concentration in soybean (*Glycine max* L. Merr.) (Eskandari et al., 2013). Subsequently, because of the multiallelism and higher level of polymorphism at simple sequence repeat (SSR) loci than at other types of markers, a genome-wide association study (GWAS) has successfully been carried out in a few studies using SSR markers. Breseghello and Sorrells (2006) found that 14 and 6 SSR markers were associated with kernel size and milling quality in a collection of hexaploid winter wheat from the eastern USA. Wang et al. (2012); Zhang et al. (2012); Guo et al. (2015), and Yao et al. (2009) detected dozens of SSR loci associated with yield-related traits using different association populations. Kollers et al. (2013) identified 794 significant marker-trait associations (MTAs) involving 323 SSR alleles controlling *Fusarium* head blight resistance in a panel of 372 European wheat varieties. However, the density of markers in these association studies was relatively low.

With the rapid development of next-generation sequencing and high-density marker genotyping techniques, GWAS has become an increasingly popular and effective method for studying complex traits in various plant species such as wheat (Liu et al., 2017; Sun et al., 2017), maize (Tian et al., 2011), rice (*Oryza sativa* L.) (Wu J. et al., 2015), soybean (Hwang et al., 2014), barley (Kraakman et al., 2006), *Aegilopstauschii* (Liu et al., 2015), and cotton (*Gossypium* spp.) (Gapare et al., 2017). Recently, diversity array technology (DArT) markers were used to identify associations with different traits (Yu et al., 2011; Bordes et al., 2014). For example, Crossa et al. (2007) used 813 DArT markers to identify associations with resistance to stem rust, leaf rust, yellow rust, and powdery mildew was well as grain yield in five historical wheat international multienvironment trials at the International Maize and Wheat Improvement Center (CIMMYT). Gouis et al. (2012) detected 62 markers individually associated to earliness components corresponding to 33 chromosomal regions in a 227-wheat accessions. Three hundred and eighty-five significant MTAs were reported by Neumann et al. (2011) in 96 wheat accessions. At present, high-density SNP genotyping arrays including 9K, 90K, 660K, and 820K SNP assays have been developed in wheat (Rasheed et al., 2017), and some of them have been used to perform a GWAS in a few studies. For instance, the 90K wheat SNP chip has been used to study the genetic control of yield-related traits in a panel of 66 elite wheat accessions derived from Xiaoyan6 and identify a total of 803 significant MTAs that explain up to 35.0% of the phenotypic variation (Ma et al., 2018). A GWAS of 163 bread wheat cultivars from the Yellow and Huai Valley of China using the 90K SNP genotyping chip identified dozens of loci controlling 13 agronomic traits that explained approximately 20% of the phenotypic variation on average (Sun et al., 2017). Four QTLs for grain colour and 12 QTLs for preharvest sprouting resistance were identified in at least two environments in a panel of 185 U.S. elite breeding lines and cultivars using the wheat 9K and 90K SNP arrays (Lin et al., 2016). Recently, a newly developed wheat 55K SNP genotyping assay was designed by the Chinese Academy of Agricultural Sciences based on the wheat 660K SNP array, and it is genome-specific with higher polymorphism and informativeness and is highly cost-effective with a wide range of potential applications.

China is the largest wheat producer in the world, and the annual wheat production shares total approximately 20% of global wheat production (He, 2001). Recently, GWASs were performed for yield-related traits in 163 wheat cultivars from the Yellow and Huai Valley of China (Sun et al., 2017) and for quality-related traits in 192 wheat lines from southwest China using the 90K SNP genotyping chip, for agronomic traits in Chinese wheat landraces using DArT markers (Liu et al., 2017), and for phosphorus-deficiency tolerance traits in *Aegilopstauschii* using a 10K SNP array (Liu et al., 2015). In this study, a GWAS for yield and yield-related traits in 15 environments across five main agro-ecological zones was performed in a diverse panel of 384 wheat cultivars and excellent germplasm collections from several major wheat ecological areas by using the newly developed wheat 55K genotyping assay. Our main objectives were to identify some major stable SNPs with superior alleles for yield-related traits and to investigate the genetic difference between founder parents and widely planted cultivars. This study is useful for marker-assisted selection for yield improvement and dissecting the genetic mechanism of important cultivars in wheat.

## Materials and Methods

### Plant Material and Phenotyping

A set of 384 diverse wheat cultivars was used in this study (**Table S1**). Three hundred and thirty-eight of them originated from eleven Chinese provinces, namely Hebei (65), Henan (62), Jiangsu (56), Shaanxi (53), Gansu (23), Shandong (20), Sichuan (15), Shanxi (12), Hubei (12), Heilongjiang (10), and Fujian (10). Twenty foreign varieties were also included. These cultivars included 18 founder parents and 15 widely grown cultivars with annual maximum acreages of over 667,000 ha (Zhuang, 2003). These accessions were planted in randomized complete blocks with two or three replicates in five major wheat ecological regions, including Yangling (108.08°E, 34.27°N) in Shaanxi Province, Tai′an (117.09°E, 36.21°N) in Shandong Province, Shijiazhuang (114.52°E, 38.05°N) in Hebei Province, Chengdou (104.08°E, 30.66°N) in Sichuan Province, and Yangzhou (119.42°E, 32.40°N) in Jiangsu Province in the 2007, 2008, and 2009 planting seasons. Each plot consisted of 200 plants that were grown in ﬁve rows 2 m long and spaced 30 cm apart. Ten plants from the centre of each plot were measured to investigate the following traits: plant height (PH), spike length (SL), fertile spikelet number per spike (FSNS), total spikelet number per spike (TSNS), kernel number per spike (KNS), spike number per plant (SNPP), 1,000-kernel weight (TKW), and grain weight per spike (GWS). The yield (YD) of each plot was also surveyed. However, not all the traits were recorded in all environments (**Table S2**). Broad sense heritability was calculated based on the ANOVA model as described by Ma et al. (2018).

### SNP Genotyping

Genomic DNA of each accession was extracted using the method from Saghai-Maroof et al. (1984). The DNA was genotyped with the high-density Illumina Infinium iSelect 55K SNP array. Markers with a minor allele frequency of less than 5% or showed more than 10% missing values were removed from the data set. Finally, 50,374 SNP markers were used for association analysis.

### Population Structure

Population structure was characterized using 8,000 SNPs distributed evenly on 21 wheat chromosomes in STRUCTURE2.3.4 (Pritchard et al., 2000). The number of presumed subpopulations (K) was 2 to 19 and an admixture model and correlated allelic frequencies were assumed. Ten runs of STRUCTURE were performed with 50,000 replicates for a burn-in and 10,000 replicates for Markov chain Monte Carlo (MCMC) analysis. The number of subpopulations and the best output were determined following the ΔK method (Evanno, 2005). A neighbour-joining tree was also constructed by TASSEL3.0 software.

### Association Study

Association analysis was conducted between SNP markers and phenotypic data in individual environments and best linear unbiased prediction (BLUP) values across all environments with TASSEL3.0 software. The mixed linear model (MLM) with population structure and kinship coefﬁcients was applied. The threshold for the P value (1.98 × 10^-5^) was calculated based on the number of the markers (*P* = 1/n, n = total number of SNPs used) according to the method reported by Li et al. (2013). For multiple comparison adjustment, false discovery rate (FDR)-adjusted P values (q values) were also calculated for each trait (Storey, 2002).

## Results

### Agronomic Traits and Population Structure

The investigated traits in the association population showed abundant variation in the surveyed environments, as shown in **Table S3**. The average CV of all traits across all environments was 0.16, indicating that trait values differed between cultivars. The Pearson’s correlation coefficients based on the average values across all environments for the eight agronomic traits and yield ranged from 0.056 to 0.919 (**Table S4**). SNPP was negatively correlated with all other traits except for PH. KNS and GWS simultaneously showed positive correlations with SL, YD, and TSNS. Broad sense heritability of the eight traits and yield ranged from 0.77 for YD to 0.98 for PH.

**Figure 1:**
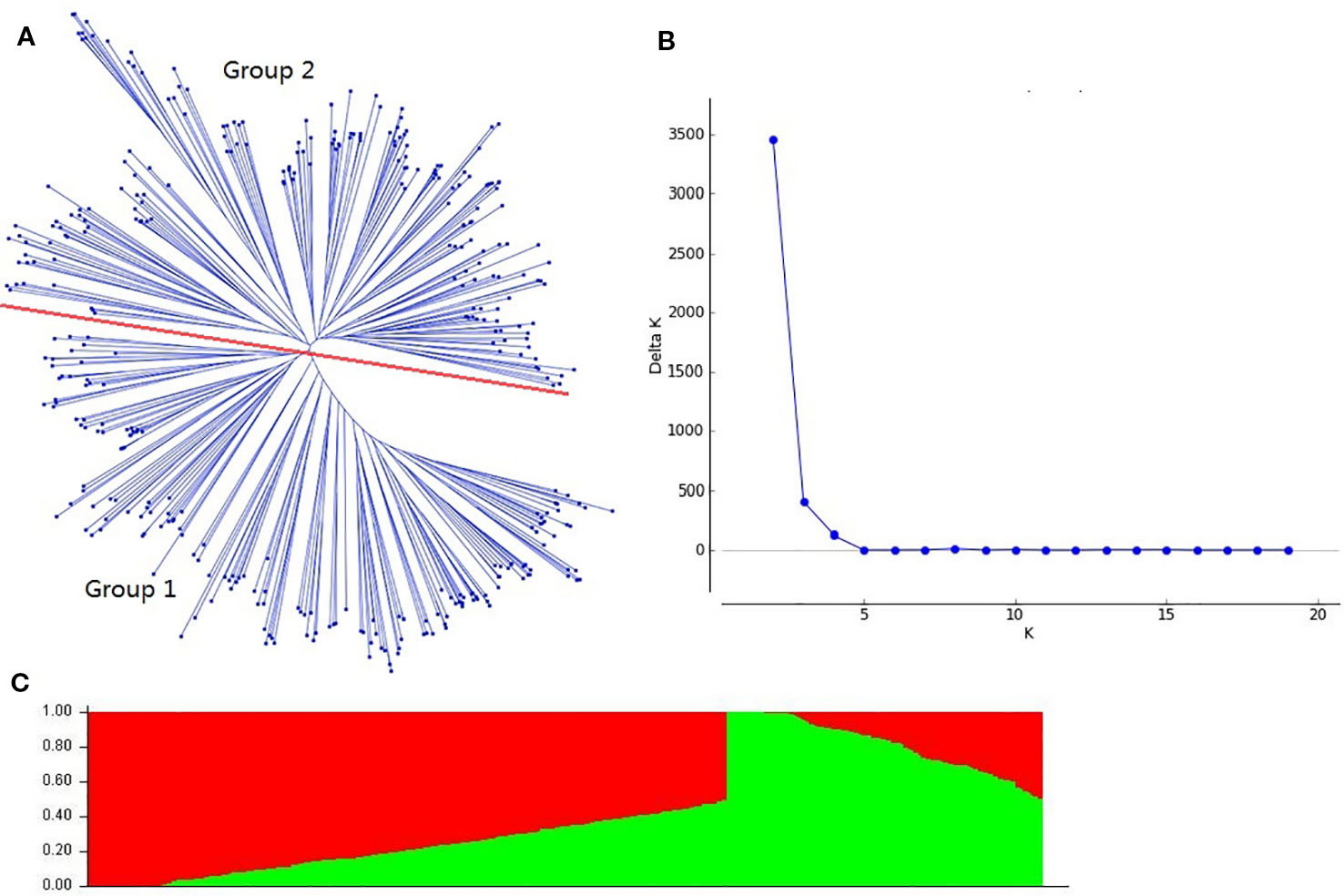
Population structure of 384 wheat accessions based on 8,000 single nucleotide polymorphism (SNP) markers across whole genome. **(A)**: Neighbor-joining tree of the 384 accessions; **(B)**: Number of subpopulations estimated by delta K; **(C)**: Genetic structure produced by the STRUCTURE software.

The cluster analysis showed that the genotypes were classified into two groups (**Figure 1A**), which was also supported by the structure analysis. The number of subpopulations (k) was plotted against the delta k calculated from STRUCTURE software. The peak of the broken line graph was observed at k = 2, indicating that the 384 accessions could be divided into two subpopulations (**Figures 1B, C**). The group I (257) was composed of eight founder parents (Funo, Xiaoyan6, Fan6, Abbondanza, Nanda2419, Wuyimai, Orofen and Aimengniu), and most of the remaining accessions were their derivatives. The group II (127) contained 10 other founder parents used in this study, and the remaining accessions were dominated by the lines with Beijing8, Bima4, Early Piemium, Mazhamai, Yanda1817, and Lovrin10 background.

### Marker-Trait Associations

**Figure 2:**
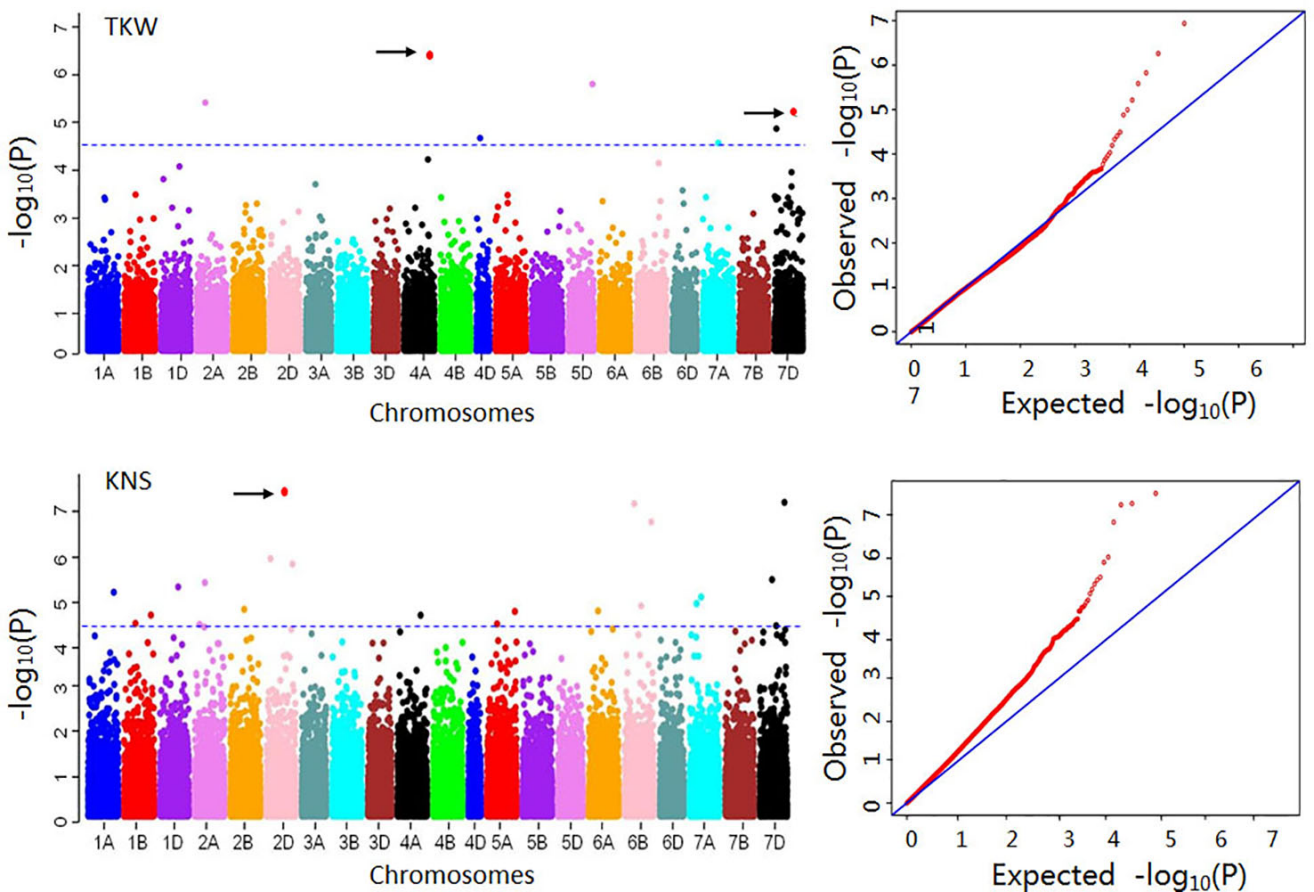
Manhattan and Q-Q plots for 1,000-kernel weight (TKW) and kernel number per spike (KNS). The blue horizontal line indicates threshold for significance. For TKW, the red dots on 4A and 7D represent markers AX-111600193 and AX-108838800, which showed positive effects in 12 and 10 environments, respectively. For KNS, the dot on 2D represents the marker AX-110982403, which showed a positive effect in 15 environments.

A total of 1,566 MTAs mapping to 460 loci were detected for eight yield-related traits using 50,374 SNPs (**Table S5**, **Figure 2**). Out of these, 19.0% of the MTAs were found only in a single environment and the remaining 81.0% were repeatedly detected in two or more environments. The phenotypic variation explained (PVE) by each marker ranged from 4.9% to 32.0%, with an average of 8.7%. The maximum number of significant SNPs was detected for SNPP (157), followed by SL (109), TSNS (107), and GWS (81). Forty-three, 41, 34, and 13 significant markers were identified for PH, FSNS, KNS, and TKW, respectively. Chromosome-wise, more significant SNPs were detected on chromosome 7D for SL, TSNS, and GWS. Distribution of the significant SNPs for eight agronomic traits on 21 wheat chromosomes was showed in **Table S6**.

### Allelic Effects on Agronomic Traits

**Figure 3:**
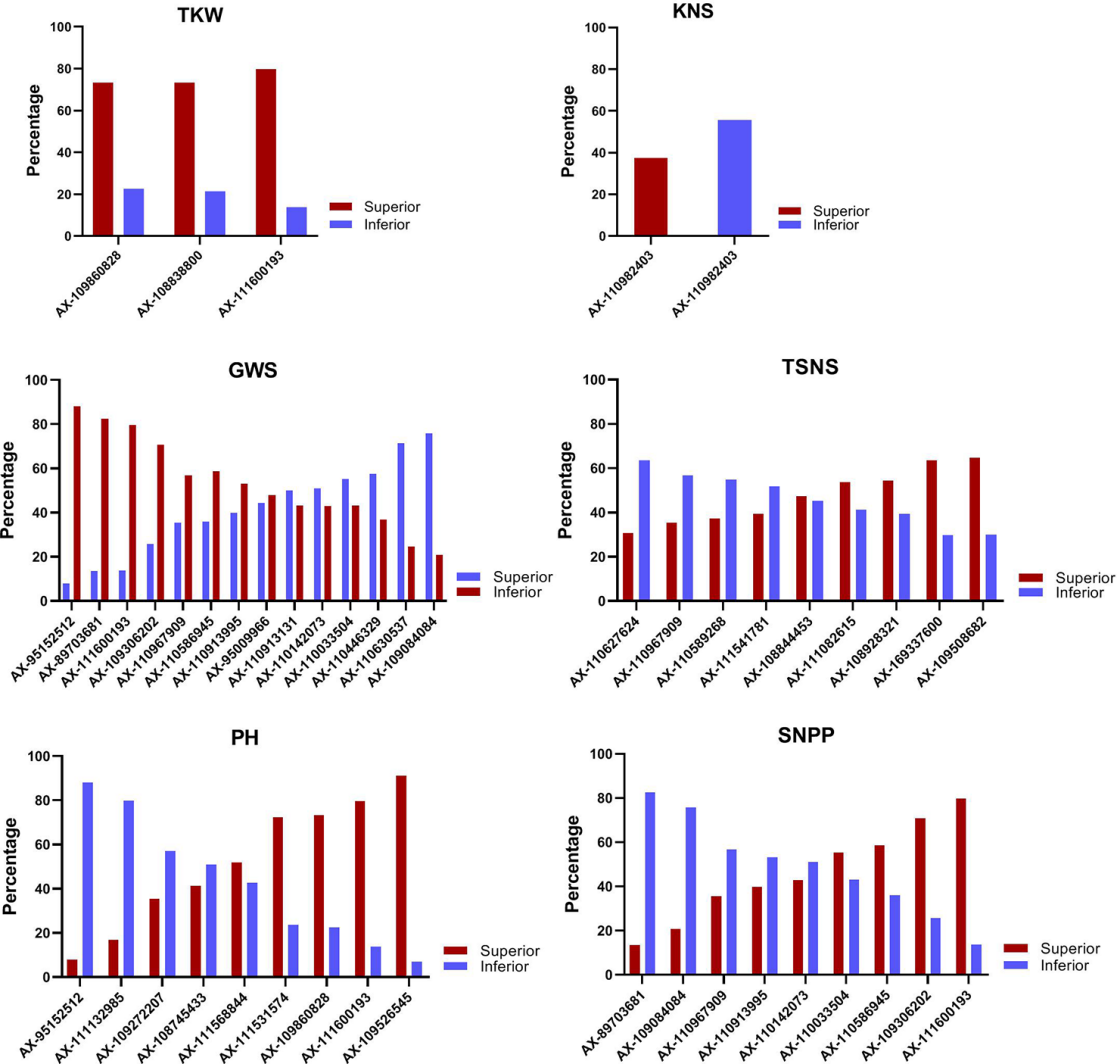
The percentage of cultivars with superior alleles at different significant loci for six traits.

The allele effects of the significant markers associated with at least two environments were estimated. The markers for which differences in phenotypic values grouped by SNP polymorphism simultaneously reached significant levels (*P* < 0.05) in nine or more environments were considered as important genetic loci in this study. Finally, a total of forty-five superior alleles mapping to 31 loci (two alleles at the same SNP locus might have showed positive effects for different traits) were selected (**Table 1** and **Table S7**). The percentages of cultivars with superior alleles at the 31 loci for the six traits were showed in **Figure 3**.

**Table 1:** Comparison of important SNP loci between this study and previous studies.

Trait	Marker	Chr	Physical position (Mb)	Loci or genes and the position (Mb) previously reported on the same chromosome	Literature
TKW	AX-111600193	4A	642.4	Xgwm160 (719.3)	Reif et al. (2011)
				Xgwm494 ~ Xgwm162 (129.3)	McCartney et al. (2005)
				*TaCWI*(607.9)	Jiang et al. (2015)
	AX-109860828	5B	422.0	Kukri_c37442_662 (353.0) ~ wsnp_Ex_c5632_9904112 (357.6)	Ma et al. (2018)
				Xbarc59 (670.7) ~ Xbarc243 (685.0)	Wang et al. (2011)
				Excalibur_c23801_115 (700.2) ~ BS00060460_51 (701.3)	Sun et al. (2017)
				Xgwm408 (577.1)	Reif et al. (2011)
				Xgwm371 (447.2)	Ramya et al. (2010)
				wsnp_Ex_rep_c66651_64962429 (403.8) ~ IAAV4074 (430.5)	Ain et al. (2015)
				Xgwm544 (77.8)	Huang et al. (2003)
	AX-108838800	7D	524.7	orw6	Reif et al. (2011)
				*QTkw-7D.1*, *QTkw-7D.2*	Cui et al. (2014)
KNS	AX-110982403	2D	525.9	Xcfd44 (608.6) ~ Xgwm349 (629.6)	Wang et al. (2011)
				QKnps-2D.2	Cui et al. (2014)
				Ra_c72517_981	Sun et al. (2017)
GWS	AX-110142073	3B	661.3	Xwmc527 (540.2), Xwmc326 (778.7)	Mason et al. (2010)
	AX-111600193	4A	642.4	Xpsp3028 (17.5)	Heidari et al. (2011)
	AX-110630537	4A	648.1	QKwpp-4A.1	Cui et al. (2014)
				Xbarc170 (607.9)	Mason et al. (2010)
	AX-110033504	5A	21.4	Xgwm291 (698.2), Xbarc151 (558.3)	Mason et al. (2010)
	AX-110913131	5A	702.2	Xgwm154 (21.0) ~ Xbarc180 (350.3)	Sun et al. (2010)
	AX-110586945	5B	584.7	Xwmc28 (649.5) ~ Xgwm790a	Heidari et al. (2011)
				Xgwm408 (577.1)	Golabadi et al. (2010)
	AX-95152512	5D	14.4	QKwpp-5D	Cui et al. (2014)
	AX-109084084	6B	567.7		
	AX-95009966	6D	68.1	Xgwm325 (80.0)	Mason et al. (2010)
	AX-109306202	7B	568.7		
	AX-89703681	7B	639.8		
	AX-110913995	7D	428.1		
	AX-110967909	7D	449.1		
	AX-110446329	7D	554.8		
TSNS	AX-108928321	1B	541.0		
	AX-110627624	1D	40.0		
	AX-109508682	2D	247.4		
	AX-110589268	3A	730.0		
	AX-169337600	4D		Xwmc622 (14.9) ~ Xcfd54 (503.5)	Wang et al. (2011)
	AX-108844453	5A	453.9	Xgwm126 (671.4) ~ Xgwm291 (698.2)	Wang et al. (2011)
	AX-111541781	5B	377.3	Xbarc74 (402.8) ~ Xbarc340 (42.0)	Wang et al. (2011)
	AX-111082615	7B	43.5	Xbarc278 (595.1) ~ Xbarc1181 (550.1)	Wang et al. (2011)
				Xwmc517 (651.5) ~ Xwmc311 (690.9)	Wang et al. (2011)
	AX-110967909	7D	449.1	Xcfd68 (174.8) ~ Xbarc252 (191.1)	Wang et al. (2011)
PH	AX-111531574	1B	554.5	Xgwm456 (464.9) ~ Xgwm124 (638.9)	Griffiths et al. (2012)
				Tdurum_contig27385_131	Sun et al. (2017)
	AX-111132985	2D	8.7	RAC875_c48703_148	Sun et al. (2017)
				*Rht8*	Pestsova and Roder (2002)
				Xcfd53 (23.0)	Chai et al. (2018)
				Xgwm320 (644.3) ~ 529tc	Griffiths et al. (2012)
				BE497718-260	McCartney et al. (2005)
	AX-109272207	4A	133.1	Xwmc24 (27.3) ~ Xksm130	Liu et al. (2011)
	AX-111600193	4A	642.4	Kukri_c77040_87 (625.9)	Ain et al. (2015)
	AX-108745433	4A	703.6		
	AX-109860828	5B	422.0	Xwmc289 (556.2) ~ Xbarc140 (598.0)	Griffiths et al. (2012)
				BS00009311_51	Ain et al. (2015)
				Xwmc640 (666.8)	McCartney et al. (2005)
	AX-95152512	5D	14.4	Xgwm190 (8.7) ~ Xbarc28.2	Cui et al. (2011)
	AX-111568844	7B	505.4	Xgwm46 (158.1)	Huang et al. (2003)
				Xgdm36 ~ Xbarc50 (172.4), Xcau130 ~ Xgwm537 (26.8)	Liu et al. (2011)
				Xgwm333 (475.6)	McCartney et al. (2005)
	AX-109526545	7D	397.6		
SNPP	AX-110142073	3B	661.3	Xgwm264b (145.7)	Huang et al. (2003)
				Xbarc344 (710.4) ~ Xwmc291 (790.3)	Wang et al. (2011)
	AX-111600193	4A	642.4	QSnpp-4A.1	Cui et al. (2014)
	AX-110033504	5A	21.4	tplb0049a09_1302 (698.0)	Ain et al. (2015)
	AX-110586945	5B	584.7		
	AX-109084084	6B	567.7		
	AX-109306202	7B	568.7	Xbarc243 ~ Xbarc59	Wang et al. (2011)
	AX-89703681	7B	639.8		
	AX-110913995	7D	428.1		
	AX-110967909	7D	449.1		

**Figure 4:**
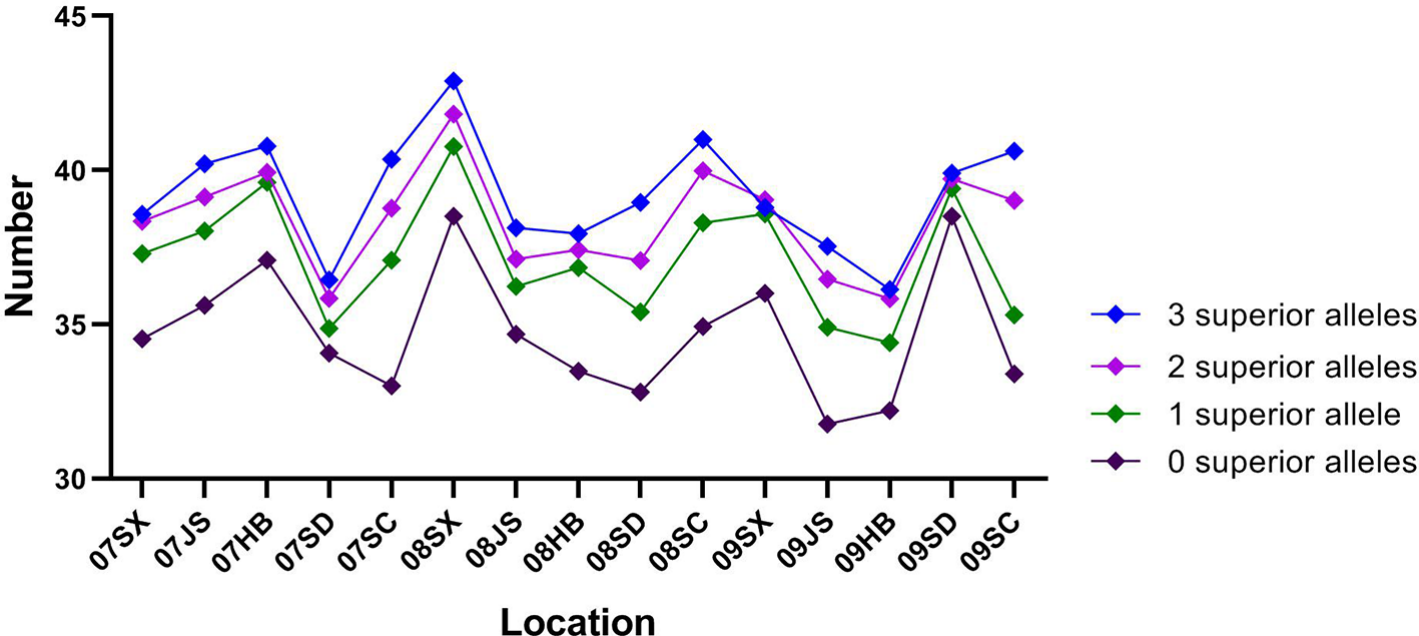
The mean 1,000-kernel weight (TKW) of cultivars with 0–3 superior alleles in 15 environments.

For TKW, three genetic loci, namely, AX-111600193, AX-109860828, and AX-108838800 on 4A, 5B, and 7D, respectively, had superior alleles. The three superior alleles showed percentages greater than 70%, implying that they had large advantages in increasing the TKW of wheat. At each of the three loci, cultivars carrying the AA allele showed a higher TKW than cultivars with the GG or CC allele in all 15 environments. The two hundred and four cultivars containing the three superior alleles showed an average TKW of 39.21 g (ranging from 36.13 to 42.90 g), and the 95 cultivars containing two of the three superior alleles showed an average TKW of 38.37 g (ranging from 35.84 to 41.82 g). In contrast, the 66 cultivars containing only one superior allele and 19 cultivars without any superior alleles displayed an average TKW of 37.14 g (ranging from 34.40 to 40.77 g) and 34.71 g (ranging from 31.76 to 38.51 g), respectively. A significant association between the number of favorable alleles and TKW was observed (r = 0.971, p = 0.029). The average TKW in cultivars with a varying number of superior alleles were showed in **Figure 4**.

For KNS, only one marker, AX-110982403 on 2D, showed a positive effect. One hundred and forty-four (37.5%) of all the used accessions contained the superior TT allele at this locus and showed an average KNS of 54.12 (ranging from 35.28 to 59.93), while 214 accessions with the inferior GG allele showed an average KNS of 45.90 (ranging from 31.51 to 54.65), suggesting that more attention should be paid to this locus to improve KNS.

For TSNS, nine markers had superior alleles across 14 environments surveyed, which were located on 1B, 1D, 2D, 3A, 4D, 5A, 5B, 7B, and 7D. Three accessions (Wilhelmina, Kehan2 and Long90-05634) contained all nine superior alleles and showed an average TSNS of 22.88 (ranging from 19.51 to 26.67), whereas the five accessions without any superior alleles showed an average TSNS of 20.85 (ranging from 16.66 to 23.78). Most cultivars (68) had five superior alleles, followed by 65, 65, 53, and 50 cultivars with 6, 4, 3, and 1 superior alleles, respectively. The number of favorable alleles in each accession was approximately correlated with TSNS (r = 0.614, p = 0.059). At four (AX-110627624, AX-110967909, AX-110589268, and AX-111541781 on 1D, 7D, 3A, and 5B, respectively) of the nine loci, cultivars with superior alleles showed a percentage of less than 40% in the accessions surveyed, indicating that they should be paid more attention in the improvement of TSNS.

For GWS, fourteen markers have superior alleles across 15 environments surveyed, which were located on 3B, 4A, 5A, 5B, 5D, 6B, 6D, 7B, and 7D. The interval between markers AX-111600193 and AX-110630537 on 4A was only 5.76 Mb. Between markers AX-110913995 and AX-110967909 on 7D, the physical distance was only 21.02 Mb. The four cultivars (Zhengzhou683, Longchun7, Wanya2, and E31846) containing all 14 superior alleles showed an average GWS of 2.20 g (ranging from 1.56 to 3.01 g), whereas the three cultivars without any superior alleles showed an average GWS of 1.48 g (ranging from 0.85 to 1.97 g). Most cultivars (63) had ten superior alleles, followed by 45, 43, 38, and 32 cultivars with 9, 8, 6, and 11 superior alleles, respectively. A significant association between the number of favorable alleles and GWS was also found (r = 0.988, p < 0.0001). Further analysis indicated that cultivars with superior alleles at AX-109084084 and AX-110630537 on 6B and 4A, respectively, were prevalent; however, cultivars with superior alleles at the other three loci (AX-111600193, AX-89703681, and AX-95152512 on 4A, 7B, and 5D, respectively) were very rare (< 15%), indicating that the three loci should be paid more attention in increasing GWS.

For PH, nine markers had superior alleles across 15 environments surveyed, which were located on 1B, 2D, 4A, 5B, 5D, 7B, and 7D. The twelve accessions containing all nine superior alleles showed an average PH of 96.03 cm (ranging from 84.89 to 108.67 cm), whereas the two cultivars without any superior alleles showed an average PH of 126.11 cm (ranging from 109.90 to 138.00 cm). Most cultivars (84) had seven superior alleles, followed by 80, 73, 41, and 40 cultivars with 6, 8, 5, and 4 superior alleles, respectively. A negative correlation was detected between the number of favorable alleles and PH (r = −0.941, p < 0.0001). Further analysis indicated that four superior alleles played important roles in regulating the PH of cultivars due to their percentage of more than 70% in the accessions surveyed. However, cultivars with superior alleles at AX-95152512 and AX-111132985 on 5D and 2D, respectively, were very rare (< 20%), indicating that they should be further applied to decrease PH in wheat breeding.

For SNPP, at nine of the 31 superior loci, there was a negative relationship between the SNPP effect versus the effect on the traits mentioned above, i.e., the alleles associated with an increased SNPP were always related to a decreased GWS, TSNS, or TKW, or an increased PH. The two cultivars (Mazhamai and Yutong286) containing all nine superior alleles showed an average SNPP of 10.52 (ranging from 4.53 to 15.66), whereas the seven cultivars containing eight of the nine superior alleles showed an average SNPP of 11.89 (ranging from 5.25 to 22.46). Forty-three cultivars without any superior alleles showed the minimum of 8.02 (ranging from 4.61 to 12.42). Most cultivars (72) had three superior alleles, followed by 66, 56, and 55 cultivars with 2, 4, and 1 superior alleles, respectively. Similarly, the number of favorable alleles in each accession was correlated with SNPP (r = 0.899, p = 0.0004). The percentage of cultivars with the superior allele of AX-89703681 was only 13.5% in the accessions surveyed, indicating that this locus should be paid more attention in improving the SNPP, whereas the opposite effect on other traits should be taken into account.

**Figure 5:**
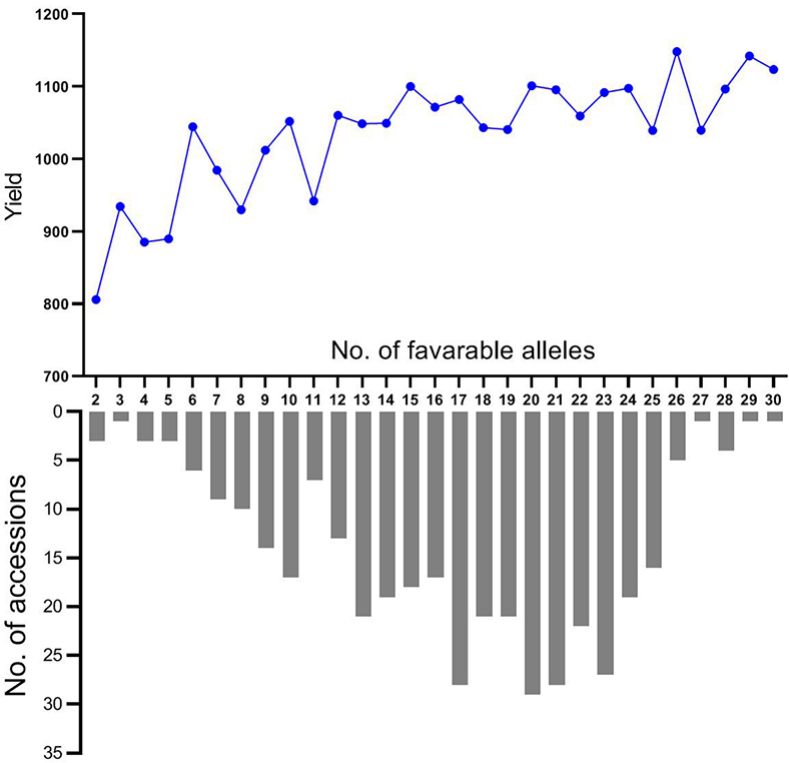
Mean yield and the number of accessions with a varying number of favorable alleles at thirty-one loci.

In general, the favorable alleles in each accession ranged from 2 to 30 with an average of 16 at the thirty-one loci in this association panel. The favorable alleles in 74.5% of all cultivars varied from 13 to 25 (**Figure 5**). The cultivar Zhengzhou683 had the largest number of favorable alleles (30) followed by Suzhou7829 (29). Each of four cultivars (Longchun7, Ningmai6, Yunmai35, and Zhen7630) had 28 favorable alleles. The cultivars with more than 20 favorable alleles and their traits were showed in **Table S8**. Moreover, mean yields of accessions were gradually increased with a varying number of favorable alleles at thirty-one loci (**Figure 5**). The accessions that contained only two favorable alleles had a mean yield of 805.96 g, whereas those accessions containing more than 27 favorable alleles had an average of 1,120.63 g. A significant association between the number of favorable alleles and yield was observed (r = 0.799, p < 0.0001), suggesting that pyramiding multiple QTL with marker-assisted selection may effectively increase yield of wheat.

### Pleiotropic Effects Revealed by GWAS

Eleven of the 31 markers were found to be associated with more than one trait based on GWAS. At four of these loci, the superior alleles showed a positive effect for two or three traits. That is, the superior AA allele of AX-111600193 on 4A was simultaneously associated with an increased TKW and GWS, and a decreased PH. The TT allele at AX-110967909 on 7D was simultaneously associated with increased GWS and TSNS. The superior AA allele of AX-109860828 on 5B regulated a high TKW and short PH, and the superior CC allele of AX-95152512 on 5D regulated a high GWS and short PH. However, the alternative GG allele at AX-111600193 and TT allele at AX-110967909 increased the SNPP. In addition, seven markers (AX-109084084, AX-109306202, AX-110033504, AX-110142073, AX-110586945, AX-110913995, and AX-89703681) were associated with increased GWS, whereas all of them showed negative allelic effects on SNPP.

### Distribution of Superior Alleles in Founder Parents and Widely Grown Cultivars

The phenotypic variation in the eight surveyed traits and yield was compared between 18 founder parents and 15 widely grown cultivars (**Table 2**). Significant differences were found between the groups of cultivars for all traits except for SL and YD. Widely grown cultivars displayed a higher TKW and GWS and shorter PH than founder parents, whereas founder parents showed a greater TSNS, KNS, and SNPP than widely grown cultivars.

**Table 2:** The phenotypic variation in eight traits and yield between founder parents and widely grown cultivars.

Trait	Widely grown cultivar			Founder parent		
	Min	Max	Mean	Min	Max	Mean
PH	60.00	173.72	100.94 ± 24.53^**^	68.20	146.00	109.01 ± 19.00
SL	5.51	21.00	9.25 ± 2.08	5.50	15.95	9.04 ± 1.55
TSNS	11.20	28.33	19.53± 2.71^**^	8.50	25.53	20.13 ± 2.35
FSNS	13.00	26.00	18.20 ± 2.08*	13.00	24.30	18.69 ± 2.04
KNS	20.55	94.50	45.04 ± 9.70^**^	13.18	86.50	48.03 ± 11.78
TKW	18.49	55.00	39.51 ± 5.30^**^	18.00	53.00	34.95 ± 6.63
SNPP	3.87	22.30	9.61 ± 3.96^*^	4.40	31.03	10.39 ± 4.64
GWS	0.58	3.96	1.82 ± 0.49^**^	0.28	3.34	1.71 ± 0.53
YD	386.67	1838.42	1061.77 ± 357.18	228.66	1796.67	1000.45 ± 359.41

TKW, 1000-kernel weight; KNS, kernel number per spike; TSNS, total spikelet number per spike; FSNS, fertile spikelet number per spike; GWS, grain weight per spike; PH, plant height; SNPP, spike number per plant; YD, yield. *P < 0.05, **P < 0.01.

**Figure 6:**
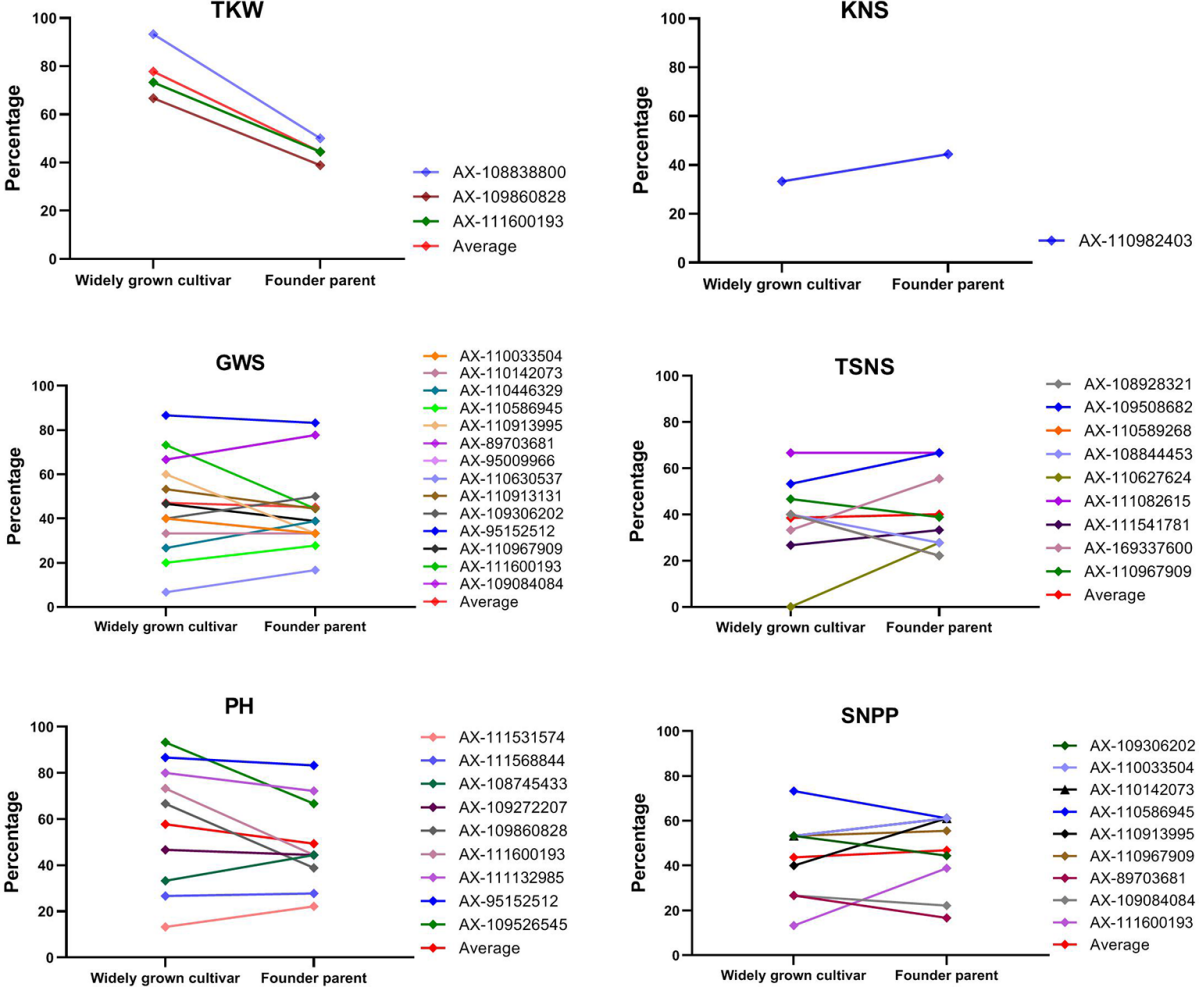
The percentage of cultivars with superior alleles at different significant loci for six traits in widely grown cultivars and founder parents.

**Figure 7:**
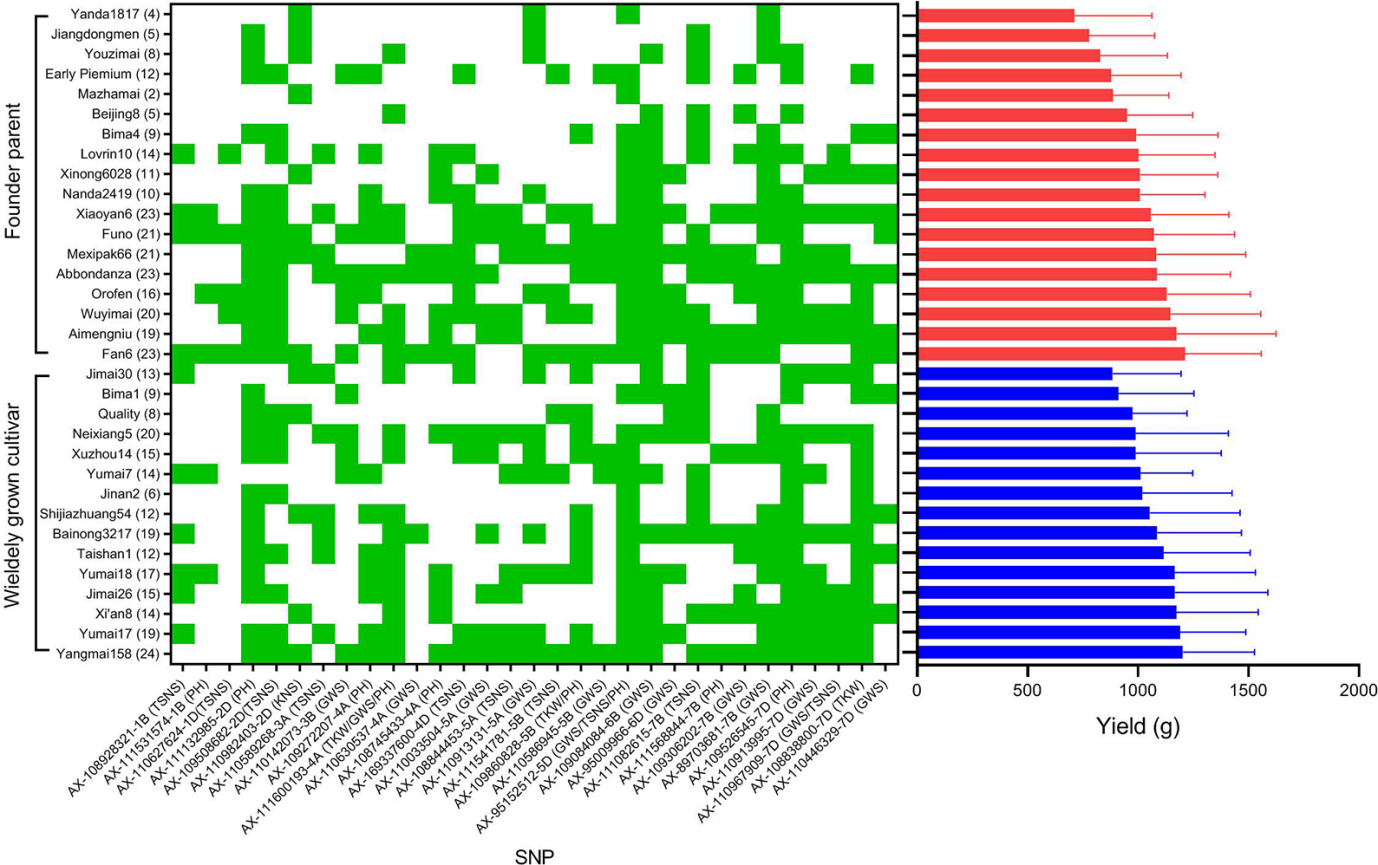
The distribution of superior alleles in widely grown cultivars and founder parents and the variation of yield. Green indicates the distribution of favorable alleles, red and blue histogram represent the mean yield of 18 founder parents and 15 widely grown cultivars, respectively. The number of favorable alleles for each cultivar is in parentheses.

The superior alleles at the 31 loci mentioned above were compared between founder parents and widely grown cultivars (**Figure 6**). For TKW, the percentages of 3 superior alleles reached 93.3%, 66.7%, and 73.3% in widely grown cultivars, which were higher than those (50.0%, 38.9%, and 44.4%, respectively) in founder parents. For KNS, the superior allele of AX-110982403 showed a higher frequency (44.4%) in founder parents than in widely grown cultivars (33.3%). For GWS, TSNS, PH, and SNPP, the percentages of superior alleles in these two groups were diverse at different loci. In general, for GWS and PH, the average frequencies of the superior alleles in widely grown cultivars were slightly higher than those in founder parents. For TSNS and SNPP, higher proportions of superior alleles were detected in founder parents than in widely grown cultivars. Overall, the percentage (49.7%) of superior alleles in widely grown cultivars was slightly higher than that (45.2%) in founder parents across all 31 loci. The distribution of superior alleles and their yield in widely grown cultivars and founder parents were showed in **Figure 7**.

**Figure 8:**
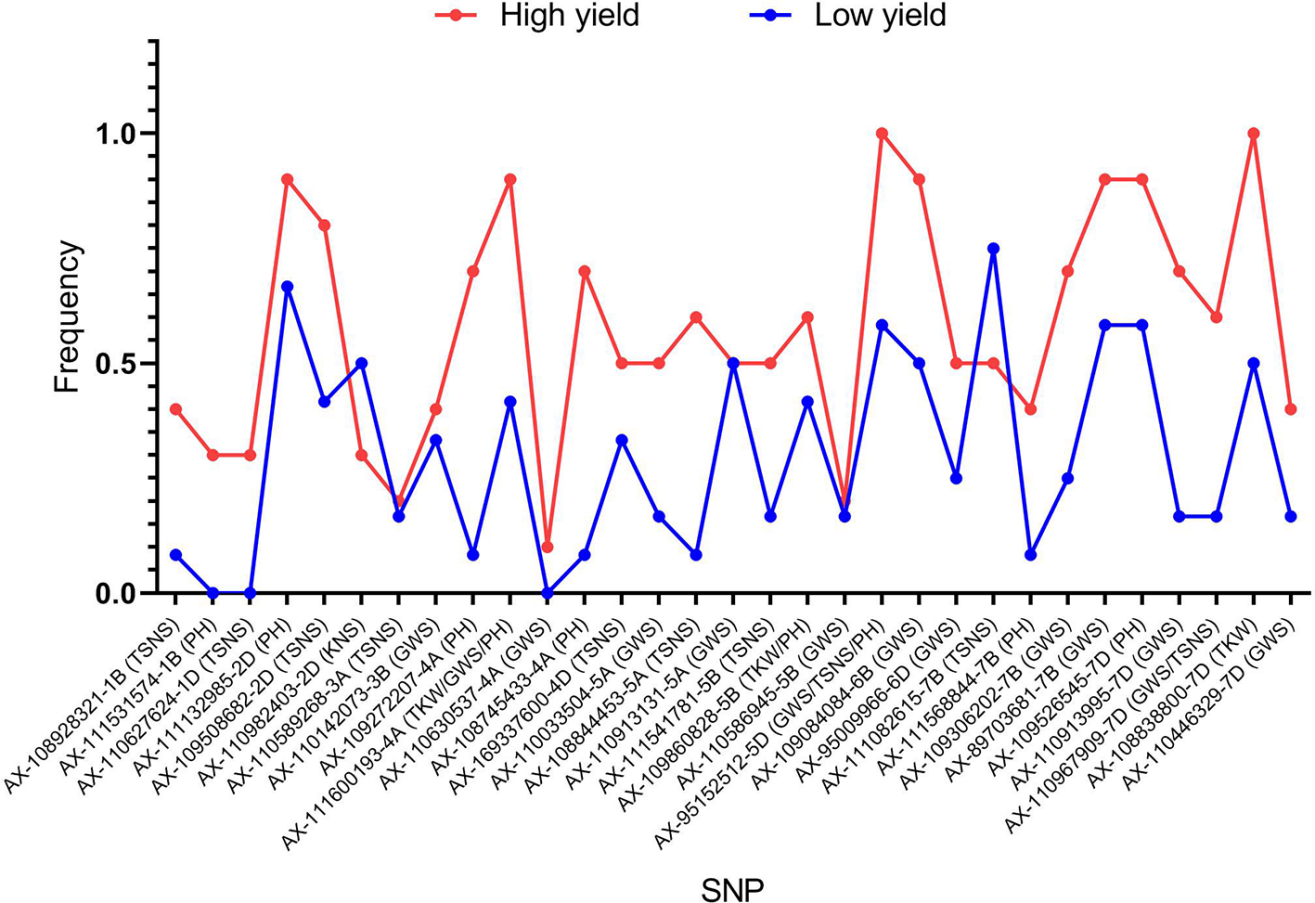
Frequencies of favorable alleles in the high yield accessions and the low yield accessions across all 31 loci.

To illustrate the important role of the 31 superior loci for yield in founder parents and widely grown cultivars, ten high yield accessions (mean yield > 1,100 g) and 10 low yield accessions (mean yield < 1,000 g) were selected. In general, the frequencies of favorable alleles in the high yield accessions were higher than in the low yield accessions across all 31 loci except AX-110982403 and AX-111082615 (**Figure 8**). Of the 31 superior loci, 15 had a frequency equal or higher than 0.60 in the high yield accessions, indicating that they possibly play an important role in modulating yield of wheat. However, the frequencies of superior alleles at only two loci (AX-111132985 and AX-111082615) were higher than 0.60 in the low yield accessions, indicating that they are important to increase wheat yield in these accessions.

## Discussion

### Population Construction and Structure

In this study, the population panel included 384 accessions from more than eleven Chinese provinces. By comparison, the sample size used in our study is larger than used in the previous studies (Liu et al., 2017; Sun et al., 2017). The evaluation of yield-related traits in 15 environments also showed wide variations. These results indicated that this population panel is suitable for association analysis of quantitative traits. Additionally, the evaluation of population genetic structure is a prerequisite of genome-wide association studies to reduce false associations between markers and traits. The cluster and model-based analysis in STRUCTURE affirmed the presence of two subpopulations in the population panel. In addition, in this study, the MLM approach, which takes into account population structure and genotype relationship and is widely used in association mapping in previous studies, could effectively control false positives (Edae et al., 2014).

### Discovery of Superior Alleles and Applications in Wheat Breeding

GWAS is an important tool for detecting favorable alleles for various traits in many plants (Rafalski, 2010). In this study, the diversity panel used was planted in five major wheat ecological regions of China over three consecutive years. The phenotyping data obtained here improved the accuracy in identifying stable genomic regions controlling interesting traits. The association study resulted in the identification of a large number of MTAs; however, the major focus of the discussion is on the identified superior alleles which may play a key role in modulating the agronomic traits of wheat cultivars (**Table 1**). Our study identified thirty-one superior allele loci, particularly the superior alleles at four loci (AX-95152512, AX-109860828, AX-111600193 and AX-110967909), each of which simultaneously showed a positive effect for two or three traits. The potential molecular functions of the signiﬁcant markers need be researched.

Uncovering superior allele loci were beneﬁcial to pyramid breeding. Although the associated markers may individually explain minor phenotypic variance, the assembling of favorable alleles from different marker loci into one recipient parent can exhibit much larger effects and may result in elite cultivars (Cai et al., 2014). Indeed, our study also found that the number of favorable alleles in each accession was significantly associated with TKW, GWS, TSNS, PH, and yield, respectively. Therefore, the superior alleles for yield-related traits should be integrated properly by marker-assisted selection in order to increase wheat yield. Additionally, in the present study, a total of 124 accessions were found, each of which carried more than 20 favorable alleles. Of these, the cultivar Zhengzhou683 had as much as 30 favorable alleles. The agronomic traits of these accessions were also showed in **Table S8**, and they can be selected as parents in wheat breeding.

### Marker-Trait Associations and Comparison With Previous Studies

In the present study, the GWAS identified only six significant markers associated with TKW in at least two environments. Three of these loci mapping on 4A, 5B, and 7D showed a positive effect. By BLAST-searching against the genome sequence from Chinese Spring wheat (IWGSC V1.0) (http://www.wheatgenome.org/), the significant SNPs detected in our study were compared with those reported previously. The three wheat chromosomes were found to harbour factors affecting TKW. In the current study, AX-111600193, which was associated with TKW, was located on 4A at position 642.4 Mb. Jiang et al. (2015) reported a TKW-associated gene, *TaCWI*, on 4A at position 607.9 Mb, and the physical distance between the *TaCWI* gene and AX-111600193 was 34.5 Mb. Reif et al. (2011) identified an SSR locus, Xgwm160, related to TKW on 4A at position 719.3 Mb. McCartney et al. (2005) identified a TKW-associated gene, *QGwt.crc-4A*, near position 129.3 Mb. In our study, AX-109860828, which was associated with TKW, was located on 5B at position 422.0 Mb. Ramya et al. (2010) and Ain et al. (2015) identified one SSR locus, Xgwm371, related to *QTkw.ncl-5B.2* and four SNPs (between wsnp_Ex_rep_c66651_64962429 and IAAV4074) associated with TKW on 5B at position 447.2 Mb and in the interval 403.8–430.5 Mb, respectively, and the physical distances between AX-109860828 and *QTkw.ncl-5B.2* and between AX-109860828 and the SNP IAAV4074 were only 25.2 Mb and 8.5 Mb, respectively. In addition, Wang et al. (2011) and Sun et al. (2017) identified a QTL and three significant SNPs associated with TKW in the interval 670.7–685.0 Mb and near 700.8 Mb on 5B, respectively. Groos et al. (2003); Reif et al. (2011) and Cui et al. (2014) also identified genomic regions related to TKW on 5B. Similarly, our results indicated that AX-108838800, which was related to TKW, was located on 7D at position 524.7 Mb, and some QTLs linked to TKW were previously detected on 7D (Reif et al., 2011; Cui et al., 2014).

Earlier studies reported eight well-known reduced height (*Rht*) genes, namely, *Rht-B1b*, *RhtD1b*, *Rht4*, *Rht5*, *Rht8*, *Rht9*, *Rht12*, and *Rht13* on chromosomes 4BS, 4DS, 2BL, 3BS, 2DS, 5AL, 5AL, and 7BS, respectively (Ellis et al., 2005). In this study, a total of 118 MTAs mapping to 43 loci, which were distributed on all chromosomes except 2A and 3D, were detected for PH in 15 environments. Nine of these loci showed a positive effect, and some were previously reported to have significant effects on PH. For example, AX-111132985, which was significantly related to PH and located on 2D at position 8.7 Mb in this study, may be linked to the *Rht8* dwarfing gene, because the marker Xgwm261 linked to the gene *Rht8* has a chromosome location (19.6 Mb) near that of AX-111132985. In addition, Chai et al. (2018) detected two novel QTLs linked in the coupling phase on 2DS with pleiotropic effects on PH and SL in the same genomic interval as *Rht8*. Other QTLs for PH were also reported on 2D in previous studies (McCartney et al., 2005; Griffiths et al., 2012; Sun et al., 2017). Nevertheless, further research is needed to identify whether these genes are identical. Similarly, in this study, AX-111531574 was found to affect PH and to be on 1B at position 554.5 Mb, which was consistent with the result obtained by Griffiths et al. (2012) who identified QTLs controlling PH between SSR markers Xgwm456 and Xgwm124 in the region 464.9–638.9 Mb. Ma et al. (2018) also detected a different QTL related to PH on 1B at position 111.0 Mb. In addition, a SNP (Tdurum_contig27385_131) linked to PH was also found in multiple environments on the same chromosome by Sun et al. (2017). Three markers, namely, AX-109272207, AX-111600193, and AX-108745433 on 4A, were associated with PH in this study. At a similar location to AX-111600193 (642.4 Mb), marker Kukri_c77040_87 (625.9 Mb) on 4A affecting PH was reported by Ain et al. (2015). Similarly, marker AX-95152512, which was related to PH, was identified on 5D at position 14.4 Mb in our study, and a QTL controlling PH at a nearby position was reported by Cui et al. (2011). Marker AX-111568844, which was related to PH, was identified on 7B at position 505.4 Mb in this study. McCartney et al. (2005) also reported an SSR locus, Xgwm333, linked to PH on 7B at position 475.6 Mb. In addition, Huang et al. (2003) and Liu et al. (2011) also detected different QTLs related to PH on 7B.

Similarly, 14, 9, and 9 superior alleles associated with GWS, TSNS and SNPP, respectively, were found in our study, of which three (AX-110630537, AX-110586945, and AX-95009966 on 4A, 5B, and 6D, respectively), one (AX-111541781 on 5B), and one (AX-110142073 on 3B) may have corresponded to previously mapped QTL regions because they had similar chromosome locations (**Table 1**). Furthermore, some superior alleles identified in this study with significant effects on TKW, GWS, TSNS, PH, and SNPP, for which no information has previously been reported in the literature for these genomic regions, may be novel QTLs. For example, nine markers (AX-110142073, AX-111600193, AX-110033504, AX-95152512, AX-109084084, AX-109306202, AX-110913995, AX-110967909, and AX-110446329) associated with GWS were consistently identified in all 15 environments in our results. More work is needed to confirm these detected loci.

### Distribution of Superior Alleles in Founder Parents and Widely Grown Cultivars

Founder parents and widely grown cultivars have played particularly crucial roles in the improvement of wheat breeding and production worldwide. For example, more than 2,000 wheat cultivars were developed from 1949 to 2000 in China, most of which can be tracked to only 16 ancestral founder parents, and 59 of them have an annual maximum acreage over 667,000 ha (Zhuang, 2003). Previous studies have identified many QTLs associated with important traits in widely planted cultivars of crops such as wheat (An et al., 2006), maize (Ordas et al., 2009) and rice (Tian et al., 2006). Moreover, a dozen of important chromosomal regions that underwent rigorous selection during breeding and may be associated with advantageous traits have been detected in founder parents of wheat (Pestsova and Roder, 2002; Han et al., 2009; Li et al., 2012), barley (Christopher et al., 2006), soybean (Lorenzen et al., 1995), potato (*Solanumtuberosum* L.) (Simko et al., 2004) and sorghum (*Sorghum bicolor* L.) (Jordan et al., 2004). However, little information is available on the difference between founder parents and widely grown cultivars, which may be useful for future wheat improvement and breeding. In this study, the widely grown cultivars showed a higher TKW and GWS, and shorter PH than the founder parents (**Table 2**), possibly because the former had more superior alleles for these traits than the latter (**Figure 6**). Similarly, Chang et al. (2018) investigated the genetic characteristics of a founder parent (Bima4), a widely planted cultivar (Bima1) and four other sibling lines that were derived from the same cross. The authors found that the widely planted cultivar Bima1 showed a higher TKW than the founder parent Bima4, and phenotypic and genomic comparisons indicated that both Bima1 and Bima4 were superior to the remaining sibling lines. We also found that the widely grown cultivars showed higher average yields than the founder parents, although the difference was not significant between them. It may be that the percentage (49.7%) of superior alleles in widely grown cultivars was slightly higher than that (45.2%) in founder parents across all 31 loci.

## Data Availability Statement

All datasets generated for this study are included in the article/**Supplementary Material**.

## Author Contributions

XL designed the experiments, analyzed all data, and wrote and extensively revised this manuscript. XX participated in phenotype measurement and data analysis. WL, XQL and XY guided data analysis. ZR and LL guided the experiment. All authors approved the final version of the manuscript.

## Funding

This study was supported by a grant from the Natural Science Foundation of China (No. 31571752) and the Project of Henan Provincial Youth Backbone Teachers (No. 2019GGJS246).

## Conflict of Interest

The authors declare that the research was conducted in the absence of any commercial or financial relationships that could be construed as a potential conflict of interest.
