# Supplementary material for: Dissection of Superior Alleles for Yield-Related Traits and Their Distribution in Important Cultivars of Wheat by Association Mapping
Source: Front Plant Sci. 2020 Mar 2;11:175. doi: 10.3389/fpls.2020.00175 (PMC7061769; doi:10.3389/fpls.2020.00175)
Supplement: Supplementary file 1 [file DataSheet_1.pdf]

TABLE S1 Information of the 384 wheat accessions used in this study.

Accession No.	Name	Origin	Type	Accession No.	Name	Origin	Type
MY000161	Abbondanza	Italy	F	ZM016154	Yu822367(white）	Henan	
MY000898	Early Piemium	America	F	ZM016182	Yubao10	Henan	
MY001072	Funo	Italy	F	ZM016194	Xuchang26	Henan	
MY001759	Lovrin10	Romania	F	ZM016196	Huapei726	Henan	
MY002004	Mexipak66	Pakistan	F	ZM017931	Yucheng851	Henan	
MY002255	Orofen	Chile	F	ZM017939	Zhengzhou743	Henan	
ZM002798	Youzimai	Shaanxi	F	ZM017950	Xinyang1	Henan	
ZM004160	Mazhamai	Shaanxi	F	ZM017958	Yanshi4	Henan	
ZM005871	Jiangdongmen	Jiangsu	F	ZM022851	Guangyang821	Henan	
ZM008963	Beijing8	Shaanxi	F	ZM022863	Hua850512	Henan	
ZM010450	Fan6	Sichuan	F	ZM022870	Hua886531	Henan	
ZM015830	Aimengniu	Shandong	F	ZM022904	Zheng87302-0-12-A	Henan	
ZM017079	Xiaoyan6	Shaanxi	F	ZM022915	Zhengzhou871	Henan	
ZM009378	Yanda1817	Beijing	F	ZM022916	Zhengzhou872	Henan	
ZM010176	Nanda2419	Jiangsu	F	ZM022972	Yutong286	Henan	
	Wuyimai	Sichuan	F	ZM022975	Yumai10	Henan	
ZM009594	Bima4	Shaanxi	F	ZM022994	Nanzhao76144-0-6-1	Henan	
ZM009597	Xinong6028	Shaanxi	F	ZM025399	Wenmai8	Henan	
ZM022235	Yangmai158	Yangzhou	W	ZM025464	Zhengzhou9285	Henan	
ZM022978	Yumai18	Yangzhou	W		Zhengmai9023	Henan	
ZM013850	Jimai30	Shandong	W	MY003143	Wilhelmina	Holland	
ZM025984	Jimai26	Shandong	W	ZM010314	Emai6	Hubei	
ZM009523	Neixiang5	Henan	W	ZM011384	Jingguangmai	Hubei	
ZM009101	Shijiazhuang54	Hebei	W	ZM016219	Huazhong6	Hubei	
ZM009391	Jinan2	Shandong	W	ZM016330	E1161	Hubei	
ZM009405	Taishan1	Shandong	W	ZM016368	E26046	Hubei	
ZM009591	Bima1	Shaanxi	W	ZM016373	E31846	Hubei	
ZM017936	Bainong3217	Henan	W	ZM016378	E34963	Hubei	
ZM017970	Yumai7	Henan	W	ZM016503	Emai9	Hubei	
ZM022977	Yumai17	Henan	W	ZM016512	Xiangmai5	Hubei	
ZM025939	Xi'an8	Shaanxi	W	ZM016597	Xiangnong3099	Hubei	
ZM010216	Xuzhou14	Jiangsu	W	ZM016606	Xiangmai10	Hubei	
MY002455	Quality	America	W	ZM023015	JingzhouHXB7561-16	Hubei	
ZM010261	Anhui3	Anhui		ZM018051	Xiang 675-1	Hunan	
ZM010279	Anhui9	Anhui		ZM018104	Xiang 1445	Hunan	
ZM010280	Anhui10	Anhui		MY002540	Rieti 75	Italy	
ZM015308	Wo80	Anhui		MY003078	Virgilio	Italy	
ZM015479	Mengke3C	Anhui		ZM009472	Zhengyin1	Italy	
ZM015488	Ai73	Anhui			Zhengyin4	Italy	
	Wanmai18	Anhui		ZM010186	Nannongdaheimang	Jiangsu	
ZM008933	Jinghong4	Beijing		ZM010187	Zhongshan2	Jiangsu	

ZM008936	Jinghong8	Beijing	ZM010194	Ningmai3	Jiangsu
ZM008937	Jinghong9	Beijing	ZM010197	Aiganzao	Jiangsu
ZM008958	Huabei187	Beijing	ZM010211	Nanda8	Jiangsu
ZM008960	Huabei672	Beijing	ZM010217	Xuzhou15	Jiangsu
ZM008961	Beijing6	Beijing	ZM010221	Xinfeng13	Jiangsu
ZM008965	Beijing10	Beijing	ZM010228	Huaiyin69-6	Jiangsu
ZM008966	Beijing11	Beijing	ZM010240	Sumai1	Jiangsu
ZM008967	Beijing12	Beijing	ZM010241	Sumai2	Jiangsu
ZM008968	Beijing13	Beijing	ZM010242	Sumai3	Jiangsu
ZM008969	Beijing14	Beijing	ZM010243	Wangmai15	Jiangsu
ZM008975	Beijing16	Beijing	ZM010244	Wangmai17	Jiangsu
ZM008978	Jingzuo208	Beijing	ZM010245	Wangmai19	Jiangsu
ZM008979	Jingzuo210	Beijing	ZM010247	Qunzhong42	Jiangsu
ZM008982	Jingzuo236	Beijing	ZM010250	Zhen7495	Jiangsu
ZM008984	Jingzuo278	Beijing	ZM014785	Fan415	Jiangsu
ZM009014	Nongda45	Beijing	ZM014848	Ningmai6	Jiangsu
ZM009018	Nongda139	Beijing	ZM014889	Ningai8628	Jiangsu
ZM009027	Nongda183	Beijing	ZM014902	Yangmai4	Jiangsu
ZM009028	Nongda311	Beijing	ZM014926	Suyang8-1-2	Jiangsu
ZM009035	Dai179	Beijing	ZM014971	Suzhou7829	Jiangsu
ZM009042	Kedong81	Beijing	ZM014972	Suzhou7906	Jiangsu
ZM009043	Kedong83	Beijing	ZM014977	Suzhou7940	Jiangsu
ZM009052	Kechun5	Beijing	ZM014978	Suzhou7946	Jiangsu
ZM009053	Kechun14	Beijing	ZM014981	Suzhou8332	Jiangsu
ZM009057	Youmangbai2	Beijing	ZM015036	Jinling1	Jiangsu
ZM009059	Youmangbai4	Beijing	ZM015098	Xuan7	Jiangsu
ZM009063	Youmangbai15	Beijing	ZM015104	Xuzhou20	Jiangsu
ZM009068	Youmanghong7	Beijing	ZM015126	Jian37	Jiangsu
ZM009069	Youmanghong8	Beijing	ZM015139	Zhen7630	Jiangsu
ZM009070	Youmanghong18	Beijing	ZM022203	Jurong03	Jiangsu
ZM009087	Dongbaita1	Beijing	ZM022216	Ningfengxiaomai	Jiangsu
ZM013099	Fengkang1	Beijing	ZM022311	Xuzhou8785	Jiangsu
ZM013100	Fengkang2	Beijing	ZM024954	Nannongda96C076	Jiangsu
ZM013102	Fengkang4	Beijing	ZM024955	Nannongda96C181	Jiangsu
ZM013103	Fengkang5	Beijing	ZM025004	Ningmaizi32	Jiangsu
ZM013105	Fengkang7	Beijing	ZM025075	Yang92-90	Jiangsu
ZM013106	Fengkang8	Beijing	ZM025137	Yangmai9	Jiangsu
ZM013107	Fengkang9	Beijing	ZM025175	Zhenmai1	Jiangsu
ZM013108	Fengkang10	Beijing	ZM026690	Ningmai8	Jiangsu
ZM013109	Jingnong79-13	Beijing	ZM026691	Ningmai9	Jiangsu
ZM013113	Fengkang15	Beijing	ZM026711	Yangmai10	Jiangsu
ZM013239	Beijing5	Beijing	ZM026712	Yangmai11	Jiangsu
ZM013330	Nongda198	Beijing	ZM026713	Yangmai12	Jiangsu
ZM013361	Jingshuang16	Beijing	ZM026714	Yangmai13	Jiangsu

ZM013370	Jingdong1	Beijing	ZM026715	Yangmai14	Jiangsu
ZM013398	Jinghua1	Beijing	ZM026716	Yangmai15	Jiangsu
ZM013404	Jingwang9	Beijing	ZM026717	Yangmai16	Jiangsu
ZM013405	Jingchun70-5321	Beijing	ZM026718	Yangmai17	Jiangsu
ZM020970	Beijing841	Beijing	ZM026800	Suyang6	Jiangsu
ZM020971	Beijing8694	Beijing	ZM010306	Wannian2	Jiangxi
ZM020984	Jing411	Beijing	ZM024056	Fengqiang7	Jilin
ZM020985	Jing437	Beijing	ZM017837	Neixiang14	Neimeng
MY001472	Jubileina I	Bulgaria	ZM010114	Xiangnong3	Qinghai
MY001473	Jubileina II	Bulgaria	ZM023615	Gahai1	Qinghai
MY009460	448	Foreign	MY001760	Lovrin13	Romania
MY000945	Etoile de Choisy	France	MY001761	Lovrin18	Romania
ZM010362	Fufan17	Fujian	MY003379	СкоросneЛka Л-1	Russia
ZM010368	Dixiuzao	Fujian	ZM009568	Shannong17	Shaanxi
ZM010370	Baimangmai	Fujian	ZM009578	Qingchun2	Shaanxi
ZM010374	Jinmai33	Fujian	ZM009580	Zhuyeqing(white)	Shaanxi
ZM010384	Pumai1	Fujian	ZM009581	Zhuyeqing(red)	Shaanxi
ZM010392	Ningmai1	Fujian	ZM009592	Bima2	Shaanxi
ZM015503	Jia35	Fujian	ZM009595	Bima5	Shaanxi
ZM015540	Funong50002	Fujian	ZM009596	Bima6	Shaanxi
ZM015545	Funong60112	Fujian	ZM009607	Xiaoyan4	Shaanxi
ZM015557	Fufan16	Fujian	ZM009614	Pulin5	Shaanxi
ZM009802	Ganmai7	Gansu	ZM009616	Xiannong68	Shaanxi
ZM009825	Longchun7	Gansu	ZM009636	Shuangfengshou	Shaanxi
ZM009843	Weidong1	Gansu	ZM009637	Pofengchuan	Shaanxi
ZM009848	Weidong7	Gansu	ZM010319	Huamai7	Shaanxi
ZM009902	5507	Gansu	ZM010327	Jingzhou1	Shaanxi
ZM009905	Linnong1	Gansu	ZM010332	Jingzhou66	Shaanxi
ZM009906	Linnong2	Gansu	ZM010565	Fengmai13	Shaanxi
ZM009907	Linmai8	Gansu	ZM017078	Xiaoyan5	Shaanxi
ZM009908	Linnong11	Gansu	ZM017092	Xi'anshixinmai	Shaanxi
ZM009909	Linnong12	Gansu	ZM017116	Shan62(9)2-1	Shaanxi
ZM009910	Linnong13	Gansu	ZM017117	Shan62(9)10-4	Shaanxi
ZM009911	Linnong14	Gansu	ZM017138	Shan5860-19-2-2	Shaanxi
ZM009924	Linmai4	Gansu	ZM017162	Shan6801-3-1-1	Shaanxi
ZM009926	Linmai6	Gansu	ZM017165	Shan6815-0-2-3	Shaanxi
ZM009927	Linmai7	Gansu	ZM017180	Shan7587-1/81	Shaanxi
ZM009977	Zhongliang11	Gansu	ZM017206	Shannong21-24	Shaanxi
ZM010002	Xifeng9	Gansu	ZM017209	Xiannong151	Shaanxi
ZM010022	Qingxuan15	Gansu	ZM018254	Xiaoyan96	Shaanxi
ZM017265	Gan81(39)-2-7	Gansu	ZM018272	Shan76(15)9-29	Shaanxi
ZM017335	Linmai26	Gansu	ZM018273	Shan76(73)33-6(white)	Shaanxi
ZM018320	Gan(39)-2	Gansu	ZM023497	Xiaoyan168	Shaanxi
ZM018328	Gan83(yuan45)-5-4-42	Gansu	ZM023568	Shanxing336	Shaanxi

ZM023599	Pingliang32	Gansu	ZM023572	Shangluo76(57)22-0-8-7-2	Shaanxi
MY001212	Heine Hvede	Germany	ZM023574	Shangluo76(57)22-0-8-7-10	Shaanxi
ZM016876	Bimai6(qian)	Guizhou	ZM023575	Shangluo76(57)22-0-8-17	Shaanxi
ZM016945	Qianhuan2	Guizhou	ZM023577	Shangluo76(57)22-8-1	Shaanxi
ZM016946	Qianhuan3	Guizhou	ZM023578	Shangluo76(57)22-8-7-1-2	Shaanxi
ZM009099	Shijiazhuang407	Hebei	ZM023580	Shangluo76(57)22-8-7-1-8	Shaanxi
ZM009103	Shijiazhuang34	Hebei	ZM024772	Shanyou225	Shaanxi
ZM009110	Xiangyang4	Hebei	ZM024776	Shan8242-1	Shaanxi
ZM009113	Shijiazhuang72	Hebei	ZM024777	Shan8242-37	Shaanxi
ZM009114	Shipin83	Hebei	ZM024903	Shanmai150	Shaanxi
ZM009119	Shi4414	Hebei		Yuanfeng175	Shaanxi
ZM009149	Xingxuan7	Hebei	ZM025946	Xiaoyan54	Shaanxi
ZM017723	Jimai24	Hebei		Xinong979	Shaanxi
ZM021382	Jimai36	Hebei	ZM025942	Xinong2208	Shaanxi
ZM024079	Gaoyou503	Hebei	ZM015831	Lumai2	Shandong
ZM009678	Hechun12	Heilongjiang	ZM009125	Jimai23	Shandong
ZM009702	Kejin6	Heilongjiang	ZM009392	Jinan4	Shandong
ZM009708	Kehan2	Heilongjiang	ZM009393	Jinan5	Shandong
ZM009719	Kezhen	Heilongjiang	ZM009396	Jianan8	Shandong
ZM014653	Ke73-402	Heilongjiang	ZM009398	Jinan10	Shandong
ZM014678	Kefeng2	Heilongjiang	ZM009399	Jinan12	Shandong
ZM014681	Kehan9	Heilongjiang	ZM009410	Dexuan1	Shandong
ZM022052	Long90-05634	Heilongjiang	ZM009419	Changle5	Shandong
ZM022170	Hei86-30	Heilongjiang	ZM009422	Jinjing3	Shandong
ZM022172	Hei86-130	Heilongjiang	ZM009423	Luzhan1	Shandong
ZM009463	Zhengzhou6	Henan	ZM015619	Changwei18	Shandong
ZM009464	Zhengzhou15	Henan	ZM015683	Jinan14	Shandong
ZM009465	Zhengzhou17	Henan	ZM015786	54405	Shandong
ZM009466	Zhengzhou24	Henan	ZM022722	lainong8442	Shandong
ZM009469	Zhengzhou683	Henan	ZM009188	Taiyuan116	Shanxi
ZM009470	Zhengzhou722	Henan	ZM009189	Taiyuan567	Shanxi
ZM009471	Zhengzhou742	Henan	ZM009263	Weidong4	Shanxi
ZM009473	Zheng6fu	Henan	ZM009267	Weidong8	Shanxi
ZM009488	Baiquan565	Henan	ZM009364	Hanxuan10	Shanxi
ZM009492	Shicha15	Henan	ZM009365	Hanxuan1	Shanxi
ZM009494	Mengxian2	Henan	ZM009382	Taiyuan566	Shanxi
ZM009497	Xuyue6	Henan	ZM014430	Linfen10	Shanxi
ZM009502	Kaifeng10	Henan	ZM014210	Linfen5064	Shanxi
ZM009506	Kaizhong70-18	Henan	ZM014434	Jinmai20	Shanxi
ZM009508	Xinzheng1	Henan	ZM014435	Jinmai21	Shanxi
ZM009515	Yanda24	Henan	ZM001215	Shanxipingyaoxiaobaimai	Shanxi
ZM009516	Yanda25	Henan	ZM010464	Mianyang4	Sichuan
ZM009517	Yanda26	Henan	ZM010467	Mianyang62-31	Sichuan
ZM009520	Suinong3	Henan	ZM010473	Xichang177	Sichuan

ZM009524	Neixiang19	Henan	ZM010478	Xichang662	Sichuan
ZM009526	Neixiang173	Henan	ZM010480	Youyimai	Sichuan
ZM009528	Xinyang12	Henan	ZM010490	Shuwan8	Sichuan
ZM015936	Huapei64-2	Henan	ZM010492	Shuwan24	Sichuan
ZM015937	Huapei64-6	Henan	ZM010516	Wanya2	Sichuan
ZM015939	Huapei83-1	Henan	ZM016677	Chuan533	Sichuan
ZM015942	Huapei112-2	Henan	ZM016678	Chuan578	Sichuan
ZM015946	Huapei126	Henan	ZM016703	Chuan78001	Sichuan
ZM015948	Huapei128	Henan	ZM016789	Mianyang12	Sichuan
ZM015964	Zheng79201	Henan	ZM023194	Chuan80-466	Sichuan
ZM015987	Zhengzhou721	Henan	ZM010588	Rikaze4	Xizang
ZM015988	Zhengzhou741	Henan	ZM010589	Rikaze8	Xizang
ZM015989	Zhengzhou761	Henan	ZM010545	Yunmai25	Yunnan
ZM015995	Zhengzhou6811	Henan	ZM010547	Yunmai27	Yunnan
ZM016082	Yu85-2325	Henan	ZM010549	778	Yunnan
ZM016116	Yu30691-1-3	Henan	ZM010571	Nanyuan1	Yunnan
ZM016119	Yu30691-1-11	Henan	ZM016966	Yunmai35	Yunnan
ZM016120	Yu30691-3-6(white)	Henan	ZM015195	Bosu1	Zhejiang
ZM016153	Yu822367	Henan	ZM022407	Zhebaoda85pin8	Zhejiang

F, founder parents; W, widely-grown cultivar

TABLE S2 Data acquisition for 8 traits and yield from five locations in 2007, 2008 and 2009.

Full trait name	Abbreviation	2007					2008					2009				
		SX	JS	HB	SD	SC	SX	JS	HB	SD	SC	SX	JS	HB	SD	SC
Plant height	PH	Y	Y	Y	Y	Y	Y	Y	Y	Y	Y	Y	Y	Y	Y	Y
Spike length	SL	Y	Y	Y	Y	Y	Y	Y	Y	Y	Y	Y	Y	Y	Y	Y
Total spikelet number per spike	TSNS	Y	Y	Y	Y	-	Y	Y	Y	Y	Y	Y	Y	Y	Y	Y
Kernel number per spike	KNS	Y	Y	Y	Y	Y	Y	Y	Y	Y	Y	Y	Y	Y	Y	Y
Thousand kernel weight	TKW	Y	Y	Y	Y	Y	Y	Y	Y	Y	Y	Y	Y	Y	Y	Y
Spike number per plant	SNPP	Y	Y	Y	Y	-	Y	Y	Y	Y	-	Y	Y	Y	Y	-
Fertile spikelet number per spike	FSNS	-	Y	Y	Y	-	-	Y	Y	-	Y	-	Y	Y	Y	Y
Grain weight per spike	GWS	Y	Y	Y	Y	Y	Y	Y	Y	Y	Y	Y	Y	Y	Y	Y
Yield	YD	Y					Y	Y	Y	Y	Y	Y	Y	Y	Y	Y

SX, HB, SD, SC and JS refer to Shaanxi, Hebei, Shandong, Sichuan and Jiangsu locations, respectively. '–', indicated that data were missing

TABLE S3 Descriptive statistics of phenotypic variations in 8 traits and yield.

Year	Location	Item	PH	SL	TSNS	KNS	TKW	SNPP	FSNS	YD	GWS
2007	Shaanxi	Mean	102.77	9.14	19.11	51.84	38.09	13.32	-	798.05	1.97
		Max	148.20	16.70	26.60	82.20	57.80	23.90	-	1226.50	3.07
		Min	52.30	5.70	15.00	35.50	23.70	6.90	-	270.00	1.20
		Stdev	19.72	1.54	1.56	6.94	4.83	2.77	-	164.05	0.33
		CV	0.19	0.17	0.08	0.13	0.13	0.21	-	0.21	0.17
	Yangzhou	Mean	106.98	9.96	21.97	51.99	39.33	11.14	20.30	-	2.28
		Max	155.80	18.74	30.43	80.05	57.10	19.88	27.77	-	3.93

		Min	57.55	7.01	18.30	31.70	23.05	4.63	16.60	-	0.66
		Stdev	17.57	1.39	1.69	8.57	5.88	2.25	1.57	-	0.49
		CV	0.16	0.14	0.08	0.16	0.15	0.20	0.08	-	0.22
	Hebei	Mean	98.27	9.45	20.78	56.98	40.18	10.14	19.48	-	2.28
		Max	147.00	21.00	29.00	94.50	63.72	21.50	27.00	-	4.50
		Min	50.00	5.20	16.00	31.50	23.12	3.50	15.00	-	1.01
		Stdev	19.83	1.75	1.89	11.22	5.71	2.99	1.92	-	0.52
		CV	0.20	0.18	0.09	0.20	0.14	0.30	0.10	-	0.23
	Shandong	Mean	96.95	10.04	18.78	48.02	35.90	15.72	17.31	-	1.72
		Max	132.13	15.80	25.33	77.80	54.20	39.53	22.37	-	2.90
		Min	48.20	6.40	12.63	24.85	19.80	3.50	9.57	-	0.68
		Stdev	16.22	1.50	1.66	8.21	5.67	4.78	1.85	-	0.39
		CV	0.17	0.15	0.09	0.17	0.16	0.30	0.11	-	0.23
	Sichuan	Mean	112.97	8.72	-	46.28	39.02	-	-	-	1.81
		Max	155.00	16.00	-	92.00	59.00	-	-	-	4.03
		Min	55.00	5.00	-	14.00	15.00	-	-	-	0.43
		Stdev	18.61	1.51	-	13.32	6.93	-	-	-	0.62
		CV	0.16	0.17	-	0.29	0.18	-	-	-	0.34
2008	Shaanxi	Mean	99.59	8.68	19.76	51.45	42.05	6.03	-	1345.66	2.16
		Max	146.30	16.00	25.77	81.47	62.97	11.83	-	1970.73	3.22
		Min	51.14	4.92	14.37	35.13	26.37	3.37	-	733.43	1.22
		Stdev	17.42	1.42	1.72	6.38	5.00	1.20	-	175.19	0.33
		CV	0.17	0.16	0.09	0.12	0.12	0.20	-	0.13	0.15
	Yangzhou	Mean	104.75	10.03	21.17	51.67	37.37	10.05	19.64	-	2.29
		Max	145.29	17.96	27.77	77.97	56.97	15.77	25.90	-	3.83
		Min	55.83	6.34	16.90	29.10	19.03	5.53	16.00	-	0.90
		Stdev	14.94	1.51	1.56	7.76	5.88	1.72	1.39	-	0.45
		CV	0.14	0.15	0.07	0.15	0.16	0.17	0.07	-	0.20
	Hebei	Mean	90.12	9.18	19.49	50.46	37.40	7.38	18.70	1012.12	1.88
		Max	148.50	20.85	26.67	80.78	64.04	19.30	24.34	1554.77	3.12
		Min	45.77	5.70	13.33	27.56	21.69	3.97	10.34	113.93	0.69
		Stdev	17.83	1.93	1.98	9.26	5.49	1.99	1.92	245.85	0.40
		CV	0.20	0.21	0.10	0.18	0.15	0.27	0.10	0.24	0.21
	Shandong	Mean	98.56	9.49	18.49	41.02	37.56	6.50	-	1340.89	1.54
		Max	131.53	13.50	21.70	54.87	53.50	10.47	-	2000.00	2.42
		Min	56.70	7.07	15.87	28.60	21.57	4.30	-	543.33	0.92
		Stdev	16.65	0.95	1.07	5.04	6.54	1.00	-	289.04	0.34
		CV	0.17	0.10	0.06	0.12	0.17	0.15	-	0.22	0.22
	Sichuan	Mean	101.55	7.86	20.64	33.15	39.98	-	16.82	575.80	1.33
		Max	146.08	14.57	26.23	67.49	62.10	-	21.93	933.33	2.60
		Min	51.62	5.22	16.55	15.58	26.33	-	13.00	213.33	0.57
		Stdev	17.46	1.17	1.55	6.41	5.12	-	1.40	128.50	0.30
		CV	0.17	0.15	0.08	0.19	0.13	-	0.08	0.22	0.23
2009	Shaanxi	Mean	93.81	9.13	15.71	51.38	38.68	7.22	-	1097.90	1.98

	Max	139.34	16.37	23.30	79.40	53.90	12.50	-	1642.30	3.18
	Min	50.27	5.38	7.40	31.60	25.90	4.00	-	568.50	1.15
	Stdev	13.85	1.73	2.56	7.39	4.59	1.46	-	210.78	0.36
	CV	0.15	0.19	0.16	0.14	0.12	0.20	-	0.19	0.18
Yangzhou	Mean	106.95	9.99	21.03	50.72	36.52	5.06	19.29	-	1.85
	Max	157.33	17.07	29.00	81.43	55.10	8.27	28.57	-	3.20
	Min	64.97	6.49	17.80	28.40	21.83	1.95	16.10	-	0.97
	Stdev	16.66	1.48	1.43	8.98	4.97	0.95	1.28	-	0.42
	CV	0.16	0.15	0.07	0.18	0.14	0.19	0.07	-	0.23
Hebei	Mean	89.81	9.09	19.42	53.14	35.56	11.63	18.70	1433.93	1.88
	Max	130.87	19.07	25.96	88.00	52.08	28.29	24.36	2025.82	3.46
	Min	48.38	5.25	16.14	32.61	21.20	5.63	15.17	610.03	1.07
	Stdev	15.82	1.64	1.51	8.92	4.59	3.39	1.50	221.73	0.35
	CV	0.18	0.18	0.08	0.17	0.13	0.29	0.08	0.15	0.18
Shandong	Mean	103.34	8.84	19.10	42.37	39.70	7.15	17.07	1356.81	1.68
	Max	145.37	18.57	25.33	61.10	56.23	12.07	22.57	1803.33	2.64
	Min	51.73	5.93	14.43	28.40	23.70	4.20	12.33	790.00	1.01
	Stdev	20.31	1.33	1.41	5.83	4.98	1.51	1.43	184.92	0.29
	CV	0.20	0.15	0.07	0.14	0.13	0.21	0.08	0.14	0.17
Sichuan	Mean	106.40	8.38	21.15	34.80	38.94	-	16.91	653.02	1.35
	Max	173.72	12.36	28.33	61.14	76.52	-	22.56	987.28	3.32
	Min	52.31	5.83	12.74	13.18	13.53	-	8.95	160.51	0.28
	Stdev	19.35	0.95	1.61	7.44	7.89	-	1.67	144.46	0.36
	CV	0.18	0.11	0.08	0.21	0.20	-	0.10	0.22	0.26
Heritability		0.98	0.94	0.91	0.84	0.94	0.85	0.85	0.77	0.91

SL, spike length; TKW, 1000-kernel weight; KNS, kernel number per spike; TSNS, total spikelet number per spike; FSNS, fertile spikelet number per spike; GWS, grain weight per spike; PH, plant height; SNPP, spike number per plant; YD, yield

TABLE S4 Correlation coefficients of the 8 agronomic traits and yield among the 384 accessions.

	FSNS	TKW	KNS	GWS	SL	YD	TSNS	SNPP	PH
FSNS	1	-0.307**	0.582**	0.220**	0.361**	-0.099	0.919**	-0.249**	0.056
TKW	-0.307**	1	-0.127*	0.641**	-0.066	0.555**	-0.339**	-0.390**	-0.326**
KNS	0.582**	-0.127*	1	0.670**	0.288**	0.206**	0.478**	-0.589**	-0.242**
GWS	0.220**	0.641**	0.670**	1	0.178**	0.556**	0.119*	-0.738**	-0.389**
SL	0.361**	-0.066	0.288**	0.178**	1	-0.264**	0.444**	-0.221**	0.308**
YD	-0.099	0.555**	0.206**	0.556**	-0.264**	1	-0.172**	-0.241**	-0.612**
TSNS	0.919**	-0.339**	0.478**	0.119*	0.444**	-0.172**	1	-0.147**	0.138**
SNPP	-0.249**	-0.390**	-0.589**	-0.738**	-0.221**	-0.241**	-0.147**	1	0.323**
PH	0.056	-0.326**	-0.242**	-0.389**	0.308**	-0.612**	0.138**	0.323**	1

* and ** significant at the 0.05 and 0.01 levels, respectively. SL, spike length; TKW, 1000-kernel weight; KNS, kernel number per spike; TSNS, total spikelet number per spike; FSNS, fertile spikelet number per spike; GWS, grain weight per spike; PH, plant height; SNPP, spike number per plant; YD, yield

TABLE S5 Information for significant SNPs associated with 8 agronomic traits using phenotypic data in individual environments and BLUP value across all environments.

Trait	Marker	Chr	Pos	Average of P	Average of R^2	Average of q	Environment
TKW	AX-111525866	1D	263206152	1.15918E-05	0.0617	0.0974	09JS
	AX-108891065	2A	247644605	1.49205E-06	0.0728	0.0250	08SX
	AX-112288215	3D	546698610	1.56265E-06	0.0742	0.0374	07HB
	AX-111600193	4A	642368219	4.14082E-06	0.0762	0.1058	08SX, 09SX
	AX-111206016	4D	101658416	7.10291E-06	0.0651	0.1449	08SX, 09SX
	AX-109860828	5B	422030835	9.9646E-06	0.0623	0.1496	09JS, 08SC
	AX-86180093	5D	560329049	2.58749E-06	0.0732	0.0442	08SX, 07SX, 09JS
	AX-111554374	6A	84033013	2.8188E-06	0.0698	0.0519	09JS
	AX-109994561	7A	485512306	1.31821E-05	0.0610	0.0950	08SX
	AX-109429982	7B	660663186	3.09219E-06	0.0697	0.0519	09JS
	AX-86172687	7D	23495477	2.60768E-06	0.0721	0.0566	BLUP, 09JS, 07SX, 08SX
	AX-110584778	7D	195624197	3.71223E-07	0.0827	0.0187	07HB
	AX-108838800	7D	524655072	3.56577E-06	0.0686	0.0735	BLUP, 08SX
KNS	AX-111101445	1A	152814564	2.98904E-06	0.0718	0.0856	09HB
	AX-110909230	1A	552153001	5.74156E-06	0.0664	0.0289	08SC
	AX-111452123	1B	570572742	2.37367E-06	0.0714	0.0371	07SD
	AX-110941446	1B	648034988	1.96091E-05	0.0596	0.0554	08SC
	AX-94959165	1D	273817873	4.31443E-06	0.0675	0.0241	08SC
	AX-94991487	1D	492609848	6.14953E-06	0.0678	0.1549	07JS
	AX-111238502	2A	217341921	1.27733E-05	0.0641	0.0810	07SD
	AX-110927323	2A	269062196	3.41077E-06	0.0679	0.0215	08SC
	AX-108917295	2B	296475460	1.44255E-05	0.0601	0.0510	08SC
	AX-109924857	2B	766025783	3.6775E-06	0.0689	0.0371	07SD
	AX-109361764	2D	31605959	9.34079E-07	0.0778	0.0094	08SC
	AX-109838059	2D	64156091	3.01082E-06	0.0696	0.0371	07SD
	AX-110982403	2D	525904353	4.57252E-06	0.0793	0.1018	08SC, 08SX
	AX-108785278	2D	612329631	1.25507E-06	0.0741	0.0106	08SC
	AX-109957323	3A	744605488	1.93555E-07	0.0736	0.0098	07SD
	AX-108848855	3B	598123231	1.96713E-05	0.0492	0.0810	07SD
	AX-108776339	3B	709412018	4.70747E-06	0.0680	0.0395	07SD
	AX-110529591	4B	1110449	1.2908E-05	0.0627	0.0810	07SD
	AX-108931449	5A	462891588	6.1513E-06	0.0658	0.1549	07JS
	AX-108933860	5A	688441967	1.61601E-05	0.0600	0.0510	08SC
	AX-111596404	5D	558824993	1.47542E-05	0.0614	0.0810	07SD
	AX-109821048	6A	72428295	1.57132E-05	0.0610	0.0510	08SC
	AX-111554374	6A	84033013	3.40309E-06	0.0692	0.0856	09HB
	AX-111044766	6A	558978118	3.42339E-06	0.0712	0.1723	07SX
	AX-111006947	6A	609816201	1.55063E-06	0.0733	0.0371	07SD
	AX-109521536	6B	213489924	4.8716E-08	0.0940	0.0008	08SC
	AX-109307185	6B	404362390	8.83087E-07	0.0771	0.0445	08SX
	AX-109348334	6B	448463951	1.8484E-05	0.0498	0.0810	07SD

	AX-110536570	6B	457424533	1.19447E-05	0.0634	0.0461	08SC
	AX-110024963	6B	656594585	1.30134E-07	0.0874	0.0016	08SC
	AX-109308195	7A	92790790	1.05959E-05	0.0656	0.0445	08SC
	AX-110586229	7A	272925912	7.40197E-06	0.0641	0.0339	08SC
	AX-108902552	7D	241175886	2.90755E-06	0.0695	0.0209	08SC
	AX-94393306	7D	571861892	4.5355E-08	0.0945	0.0008	08SC
SL	AX-111619451	1A	180422966	1.2148E-05	0.0634	0.0762	09SD
	AX-109312262	1A	369324860	1.06284E-05	0.0626	0.0356	08JS
	AX-110979338	1A	477467390	4.3058E-09	0.1109	0.0000	07SC
	AX-111166388	1A	535329857	1.65451E-05	0.0517	0.0193	07SC
	AX-108935155	1A	549673017	1.18266E-07	0.0906	0.0048	07SX
	AX-109329289	1B	90544523	1.57452E-05	0.0601	0.0989	08SC
	AX-109330481	1B	389794980	9.05827E-06	0.0639	0.0761	09JS
	AX-109980222	1B	410320741	9.26371E-06	0.0653	0.0120	07SC
	AX-109966524	1B	512107124	1.66803E-05	0.0609	0.0336	08SD
	AX-109901032	1B	566185090	3.16441E-06	0.0699	0.0281	07SD
	AX-110627624	1D	40001986	2.14254E-07	0.0867	0.0005	07SC
	AX-110885924	1D	253988110	4.55199E-06	0.0741	0.0311	08SD, 07HB, 08JS, 08SX, 07SX
	AX-111525866	1D	263206152	1.22333E-06	0.0820	0.0055	07SC, 09SC
	AX-111946510	1D	478865288	4.13359E-07	0.0838	0.0009	07SC
	AX-111584834	1D	482206429	6.05225E-06	0.0944	0.0119	07SC, 09SC, 08SD
	AX-109878026	2A	68718264	1.13144E-05	0.0648	0.0270	07HB, 08SD, 07SD
	AX-108880421	2A	103141017	1.63523E-05	0.0605	0.0476	08HB
	AX-108891065	2A	247644605	4.62313E-06	0.0922	0.0299	07SC, 08SX, 08SC, 07JS, 08JS, 07SD, 07HB, 07SX, 09SC, 09JS, 09SD, 09HB
	AX-110930369	2B	533507696	6.17462E-06	0.0675	0.0148	07HB
	AX-108833859	2B	563059129	6.17012E-06	0.0694	0.0221	07HB, 08SD, 07SD, 08JS
	AX-109974803	2B	646893657	1.12724E-05	0.0624	0.0356	08JS
	AX-110412442	2B	651722410	5.23449E-06	0.0868	0.0185	07SC, 08JS, 07JS, 08SC, 08SX, 09SC
	AX-111064656	2B	652657237	6.44231E-06	0.0705	0.0181	07HB, 08SD, 08JS
	AX-108838533	2D	20349414	1.09476E-07	0.0907	0.0003	07SC
	AX-111772631	2D	20982836	1.13553E-06	0.0818	0.0055	07SC, 09SC
	AX-109546505	2D	85825611	3.76226E-07	0.0706	0.0063	08HB
	AX-109508682	2D	247376233	9.77949E-06	0.0545	0.0123	07SC
	AX-109823219	2D	325031531	1.29046E-05	0.0615	0.0450	09SC
	AX-111633510	3A	21360215	8.55252E-06	0.0657	0.0341	07SD
	AX-111039910	3A	23455186	1.28163E-07	0.0921	0.0003	07SC
	AX-109345729	3A	483161117	3.65507E-06	0.0693	0.0331	07SC, 09SD
	AX-111507661	3A	535929426	1.36621E-05	0.0632	0.0476	08HB
	AX-111811874	3A	568838239	1.7487E-05	0.0499	0.0979	09JS
	AX-110669775	3A	667086002	4.06617E-06	0.0772	0.0320	07HB, 08SD, 08JS, 08SX, 07SD, 09HB, 08HB, 07SX
	AX-109842977	3B	139033860	7.99599E-06	0.0659	0.0239	07HB, 08SD, 07SD, 08JS

AX-110563644	3B	400572493	8.66489E-06	0.0672	0.0118	07SC
AX-110035956	3B	755605526	1.83266E-05	0.0605	0.0341	08SD
AX-94469025	3D	22680050	1.34284E-05	0.0616	0.0844	09JS
AX-110021640	3D	555205315	9.16985E-06	0.0658	0.0283	07SD, 07HB
AX-110405004	3D	603459394	1.56425E-05	0.0601	0.0291	07HB
AX-95164655	3D	604303999	6.98874E-06	0.0645	0.0762	09SD
AX-108838761	4A	110334872	4.11242E-06	0.0713	0.0199	07HB, 08SD, 07SD, 08JS, 08JS
AX-109272207	4A	133050366	1.16446E-05	0.0535	0.0139	07SC
AX-110432225	4A	239579500	1.25199E-06	0.0773	0.0025	07SC
AX-109531016	4A	575803804	7.94942E-06	0.0658	0.0245	07HB, 08SD, 08JS, 07SD
AX-109841603	4A	615169027	4.76268E-06	0.0723	0.0095	08SX, 07SC
AX-111600193	4A	642368219	2.22638E-06	0.0998	0.0180	07SC, 09SC, 08SX, 08SC, 07JS, 08SD, 09JS, 08JS, 07SX, 09SD
AX-110024812	4B	14081501	8.92873E-06	0.0663	0.0320	08SD, 07SD, 08SX
AX-111167883	4B	406809257	1.05331E-05	0.0643	0.0231	07HB, 08SD
AX-110384011	4B	496553658	5.77775E-06	0.0675	0.0094	07SC
AX-108935259	4B	664244353	1.049E-05	0.0636	0.0341	07SD
AX-109986929	4D	138	8.47805E-06	0.0670	0.0118	07SC
AX-110430981	4D	375	6.83895E-06	0.0648	0.0431	08HB
AX-111157475	4D	455171149	5.17409E-06	0.0688	0.0195	08SD, 07HB, 08SX, 08JS
AX-109997529	5A	49435160	1.83652E-05	0.0598	0.0331	07HB
AX-108754211	5A	100710754	1.69816E-05	0.0669	0.0476	08HB
AX-108844453	5A	453892447	7.6903E-06	0.0660	0.0117	07SC
AX-109897379	5A	454825148	2.17617E-07	0.0868	0.0005	07SC
AX-109888319	5B	48565175	5.74386E-06	0.0636	0.0272	07HB, 08SD, 08JS, 09HB
AX-111043300	5B	296585755	7.14281E-09	0.1074	0.0000	07SC
AX-108956865	5B	301057494	8.16185E-06	0.0696	0.0249	07HB, 08SD, 07SD, 08JS
AX-110438555	5B	359132576	1.60378E-05	0.0618	0.0476	08HB
AX-109271155	5B	407044533	5.8254E-06	0.0658	0.0225	07HB, 07SD
AX-110712791	5B	653426784	3.45587E-06	0.0691	0.0581	09SD
AX-95152512	5D	14390791	4.56013E-09	0.1371	0.0002	07SC, 09SC, 07JS
AX-94398741	5D	462027776	8.27845E-06	0.0679	0.0229	07HB, 07SD
AX-109809706	5D	476819411	5.91153E-06	0.0678	0.0115	09SC, 07SC
AX-110639062	5D	495061297	5.90573E-07	0.0797	0.0298	09HB
AX-108898503	5D	554823716	1.78288E-06	0.0761	0.0116	07SC, 08SC, 09SC
AX-86180093	5D	560329049	2.82212E-06	0.1056	0.0186	07SC, 09SC, 08SX, 08SC, 07JS, 09JS, 07SX, 08JS, 08SD, 09HB, 09SD
AX-110932218	6A	1648056	2.74623E-06	0.0783	0.0252	07HB, 08JS, 09HB
AX-110946238	6A	18461050	1.59015E-05	0.0600	0.0476	08HB
AX-110993884	6A	557559454	8.27939E-06	0.0674	0.0284	07HB, 08SD, 07SD, 08HB
AX-89610547	6A	564585216	1.3807E-06	0.0751	0.0232	07SD
AX-111686735	6B	144960242	2.97934E-08	0.1009	0.0001	07SC
AX-109317829	6B	236329093	8.06447E-06	0.0656	0.0118	07SC
AX-109084084	6B	567656373	3.3166E-06	0.0968	0.0260	07SC, 09SC, 08SX, 08SC, 08SD,

							07SX, 09JS, 07JS
	AX-108758805	6B	643139473	4.79687E-06	0.0690	0.0176	07HB, 08SD, 08JS
	AX-111156621	6D	6011401	8.19255E-06	0.0669	0.0251	07HB, 08SD, 08JS, 08HB
	AX-110023271	7A	114087145	3.85463E-09	0.1107	0.0000	07SC
	AX-110380064	7A	136452857	2.8078E-06	0.0791	0.0115	08SD, 07HB, 08JS, 08SX, 07SD
	AX-89480189	7A	145491195	4.00924E-06	0.0675	0.0337	08HB
	AX-108810662	7A	493239957	5.21486E-06	0.0680	0.0191	08SD
	AX-108882731	7A	154593622	6.65043E-06	0.0657	0.0206	08SD
	AX-111576358	7A	485200708	1.69438E-05	0.0608	0.0365	07HB, 07SD
	AX-109336938	7B	8939649	3.39865E-06	0.0695	0.0428	09JS
	AX-94592534	7B	496271774	4.7049E-06	0.0688	0.0081	07SC
	AX-111568844	7B	505447589	2.02908E-06	0.0736	0.0038	07SC
	AX-110366783	7B	711490256	1.32446E-05	0.0614	0.0376	08JS
	AX-108911221	7D	4991311	4.94439E-08	0.0849	0.0002	07SC
	AX-111034889	7D	22777210	1.39352E-05	0.0608	0.0476	08HB
	AX-110503493	7D	40499462	1.0077E-05	0.0646	0.0124	07SC
	AX-109872520	7D	97305325	3.67878E-06	0.0688	0.0098	07HB
	AX-110907225	7D	112241770	5.89214E-06	0.0682	0.0197	07HB, 08SD, 07SD
	AX-109847755	7D	145671513	7.202E-06	0.0682	0.0222	07HB, 08SD, 07SD
	AX-109519401	7D	197304632	2.87892E-07	0.0718	0.0063	08HB
	AX-110804188	7D	373567260	6.21231E-06	0.0682	0.0098	07SC
	AX-111503444	7D	393285287	5.31997E-06	0.0786	0.0184	07SC, 08SX, 09SC
	AX-94712769	7D	393576415	7.92873E-06	0.0641	0.0762	09SD
	AX-108921665	7D	436396665	1.66226E-06	0.0725	0.0167	08HB
	AX-109095425	7D	520184143	1.22019E-06	0.0639	0.0154	08HB
	AX-108838800	7D	524655072	3.01132E-07	0.0874	0.0034	07SC, 08SX, 09JS, 08HB, 09SC, 08JS, 07JS, 08SC, 09SD
	AX-110349900	7D	528073469	9.24267E-06	0.0664	0.0265	07SC, 09SC
	AX-110362518	7D	529688251	1.80695E-05	0.0512	0.0207	07SC
	AX-111802901	7D	549281817	7.68848E-08	0.1241	0.0006	07SC, 09SC
	AX-110446329	7D	554832615	1.63784E-06	0.0643	0.0032	07SC
	AX-108770812	7D	561926300	4.6784E-06	0.0669	0.0337	08HB
	AX-110016687	7D	563051214	4.41684E-06	0.1063	0.0159	07SC, 09SC, 08HB
	AX-110479115	7D	628673559	1.31704E-05	0.0613	0.0476	08HB
GWS	AX-108762060	1A	535339729	6.65979E-07	0.0789	0.0021	09SC
	AX-110378928	1A	544594957	6.00357E-06	0.0713	0.0072	09SC
	AX-109876087	1B	373317133	8.05859E-06	0.0648	0.0220	BLUP, 07HB, 08HB, 09HB, 07SD, 08SD, 09SD, 07SC, 08SC, 09SC, 07JS, 08JS, 09JS, 07SX, 08SX, 09SX
	AX-109357181	1B	432442476	5.43241E-07	0.0797	0.0021	09SC
	AX-94958010	1D	407873959	1.38984E-06	0.0742	0.0022	09SC
	AX-112289622	1D	423857541	3.13744E-06	0.0711	0.0042	09SC
	AX-111164067	1D	457143978	7.9464E-06	0.0752	0.0087	09SC
	AX-110510923	1D	488715446	8.27985E-07	0.0763	0.0021	09SC

AX-111503398	2A	33039673	6.02356E-06	0.0691	0.0072	09SC
AX-111528573	2A	160791894	9.05175E-07	0.0758	0.0021	09SC
AX-108785539	2A	254463285	3.53755E-06	0.0681	0.0046	09SC
AX-109514485	2A	284416547	8.94436E-06	0.0647	0.0237	07SC
AX-110089394	2A	364472672	3.94944E-06	0.0570	0.0140	08SX
AX-110959136	2B	431032607	0.000018	0.0578	0.0412	BLUP
AX-111038927	2B	438977476	2.18414E-06	0.0709	0.0031	09SC
AX-108838533	2D	20349414	4.11952E-06	0.0691	0.0128	BLUP, 09SD, 07SC, 09SX, 08JS, 09SC, 07JS, 08SC, 08SX, 09JS, 09HB, 07SX, 07HB, 08HB, 08SD
AX-110819878	2D	646216182	1.53782E-06	0.0742	0.0023	09SC
AX-109298595	3A	623906130	1.32059E-06	0.0748	0.0022	09SC
AX-110020522	3A	686168545	2.4511E-07	0.0845	0.0012	BLUP, 07HB, 08HB, 09HB, 07SD, 08SD, 09SD, 07SC, 08SC, 09SC, 07JS, 08JS, 09JS, 07SX, 08SX, 09SX
AX-111652313	3A	23395096	7.85848E-06	0.0653	0.0087	09SC
AX-110186255	3A	702511881	9.30623E-06	0.0763	0.0098	09SC
AX-109518029	3B	73581220	1.49648E-05	0.0595	0.0343	07SX
AX-108975699	3B	432339429	6.19208E-07	0.0784	0.0021	09SC
AX-111074064	3B	448624171	3.67377E-07	0.0815	0.0017	09SC
AX-110379792	3B	556404770	1.72881E-06	0.0730	0.0025	09SC
AX-110142073	3B	661319698	1.89117E-07	0.0862	0.0011	BLUP, 07HB, 08HB, 09HB, 07SD, 08SD, 09SD, 07SC, 08SC, 09SC, 07JS, 08JS, 09JS, 07SX, 08SX, 09SX
AX-111502226	3B	808674143	1.52921E-05	0.0601	0.0350	09HB
AX-109445204	4A	449503269	8.33212E-07	0.0764	0.0021	09SC
AX-110649064	4A	629360415	1.24343E-06	0.0765	0.0022	09SC
AX-111600193	4A	642368219	6.02072E-21	0.2894	0.0000	BLUP, 07HB, 08HB, 09HB, 07SD, 08SD, 09SD, 07SC, 08SC, 09SC, 07JS, 08JS, 09JS, 07SX, 08SX, 09SX
AX-110630537	4A	648132716	1.43362E-05	0.0611	0.0345	BLUP, 07SD, 07JS
AX-111600426	4B	202670514	6.6767E-07	0.0775	0.0021	09SC
AX-110956540	4B	480690287	1.81592E-05	0.0697	0.0165	09SC
AX-110906507	4B	498880068	1.43582E-06	0.0732	0.0022	09SC
AX-110611733	4B	638844521	1.02364E-05	0.0637	0.0302	08SD
AX-109748035	4D	126811725	1.38324E-06	0.0768	0.0022	09SC
AX-109405031	4D	455657955	1.97151E-05	0.0582	0.0377	07SX
AX-108948329	5A	5424728	1.11945E-05	0.0626	0.0106	09SC
AX-110033504	5A	21418196	7.98161E-12	0.1468	0.0000	BLUP, 07HB, 08HB, 09HB, 07SD, 08SD, 09SD, 07SC, 08SC, 09SC, 07JS, 08JS, 09JS, 07SX, 08SX, 09SX
AX-109375676	5A	87986938	1.18885E-05	0.0631	0.0331	08SD
AX-110913131	5A	702223672	1.00193E-05	0.0636	0.0256	BLUP, 09HB, 07SX, 07JS, 07HB
AX-108965399	5B	75755638	1.22035E-06	0.0741	0.0022	09SC

AX-109053557	5B	230772130	1.80548E-05	0.0600	0.0165	09SC
AX-111130645	5B	266091663	1.45619E-06	0.0731	0.0022	09SC
AX-111763500	5B	392718723	2.14137E-06	0.0713	0.0083	09SD
AX-109312330	5B	403843374	1.64185E-05	0.0592	0.0344	07SX
AX-108869145	5B	579382749	1.11775E-06	0.0755	0.0022	09SC
AX-110586945	5B	584692291	2.23026E-06	0.0779	0.0082	BLUP, 07HB, 08HB, 09HB, 07SD, 08SD, 09SD, 07SC, 08SC, 09SC, 07JS, 08JS, 09JS, 07SX, 08SX, 09SX
AX-95152512	5D	14390791	5.96573E-16	0.2128	0.0000	BLUP, 07HB, 08HB, 09HB, 07SD, 08SD, 09SD, 07SC, 08SC, 09SC, 07JS, 08JS, 09JS, 07SX, 08SX, 09SX
AX-109985361	5D	440793779	7.86724E-07	0.0772	0.0021	09SC
AX-111145700	5D	446280843	1.07292E-05	0.0624	0.0245	09SD
AX-111953544	5D	552376367	1.76761E-05	0.0626	0.0408	07HB
AX-86180093	5D	560329049	5.40872E-17	0.2193	0.0000	BLUP, 07HB, 08HB, 09HB, 07SD, 08SD, 09SD, 07SC, 08SC, 09SC, 07JS, 08JS, 09JS, 07SX, 08SX, 09SX
AX-110720861	6A	25535895	7.83081E-06	0.0644	0.0215	BLUP, 07HB, 08HB, 09HB, 07SD, 08SD, 09SD, 07SC, 08SC, 09SC, 07JS, 08JS, 09JS, 07SX, 08SX, 09SX
AX-111558975	6A	415196444	6.15276E-06	0.0654	0.0185	BLUP, 07HB, 08HB, 09HB, 07SD, 08SD, 09SD, 07SC, 08SC, 09SC, 07JS, 08JS, 09JS, 07SX, 08SX, 09SX
AX-108828019	6A	596618565	1.34656E-06	0.0735	0.0022	09SC
AX-109084084	6B	567656373	8.76052E-11	0.1377	0.0000	BLUP, 07HB, 08HB, 09HB, 07SD, 08SD, 09SD, 07SC, 08SC, 09SC, 07JS, 08JS, 09JS, 07SX, 08SX, 09SX
AX-111832727	6B	644839261	8.96875E-06	0.0676	0.0226	09SD
AX-110375590	6B	690880940	1.24725E-06	0.0740	0.0022	09SC
AX-109193807	6D	14044251	9.68885E-06	0.0626	0.0258	08SX, 07SD
AX-109915068	6D	46871266	3.96097E-06	0.0686	0.0139	07SD, 08SX
AX-95009966	6D	68103868	6.99143E-06	0.0675	0.0182	BLUP, 07HB, 09JS, 09SD, 09SC, 08JS, 07SX, 07JS, 07SC, 08SX, 07SD, 08SD, 08SC
AX-111064790	7A	281664921	6.75395E-06	0.0698	0.0191	BLUP, 09HB, 08SD, 07HB, 08SX, 08HB, 07SD, 07JS, 07SX, 08SC, 09JS
AX-109878395	7A	583728108	1.31572E-06	0.0741	0.0047	09HB
AX-110391839	7A	608877194	6.53096E-06	0.0658	0.0076	09SC
AX-109832624	7A	647142350	6.71089E-06	0.0657	0.0178	09SD
AX-111818036	7A	705499935	6.97999E-06	0.0664	0.0185	09HB
AX-110163783	7A	720923551	1.34676E-05	0.0617	0.0324	07SD
AX-89376898	7B	9801961	1.43198E-06	0.0734	0.0022	09SC
AX-111128767	7B	137452909	1.83237E-05	0.0607	0.0485	08HB
AX-109306202	7B	568676196	1.83584E-07	0.0864	0.0011	BLUP, 07HB, 08HB, 09HB, 07SD,

							08SD, 09SD, 07SC, 08SC, 09SC, 07JS, 08JS, 09JS, 07SX, 08SX, 09SX
	AX-89703681	7B	639791862	1.03785E-05	0.0633	0.0245	09SC, 09JS, 09HB, 08SX, 08SC, 07HB
	AX-109309960	7D	104334359	7.53019E-07	0.0768	0.0021	09SC
	AX-89393548	7D	185764628	9.1714E-07	0.0757	0.0021	09SC
	AX-110045927	7D	393955537	1.61217E-05	0.0604	0.0344	07SX
	AX-110913995	7D	428054733	1.15813E-05	0.0630	0.0291	BLUP, 07HB, 08HB, 09HB, 07SD, 08SD, 09SD, 07SC, 08SC, 09SC, 07JS, 08JS, 09JS, 07SX, 08SX, 09SX
	AX-110967909	7D	449077527	1.10617E-07	0.0964	0.0007	BLUP, 07HB, 08HB, 09HB, 07SD, 08SD, 09SD, 07SC, 08SC, 09SC, 07JS, 08JS, 09JS, 07SX, 08SX, 09SX
	AX-94382077	7D	489903015	1.0039E-05	0.0632	0.0101	09SC
	AX-111802901	7D	549281817	3.79229E-12	0.1483	0.0000	BLUP, 09SC, 07HB, 09SX, 09SD, 07SX, 07SC, 08JS, 08SC, 07JS, 08SX, 08SD, 08HB, 09JS, 09HB
	AX-111175958	7D	553415365	3.89521E-06	0.0683	0.0140	07SX
	AX-110446329	7D	554832615	9.91941E-07	0.0653	0.0042	BLUP, 07HB, 08HB, 09HB, 07SD, 08SD, 09SD, 07SC, 08SC, 09SC, 07JS, 08JS, 09JS, 07SX, 08SX, 09SX
TSNS	AX-111532463	1A	227256286	1.58843E-05	0.0511	0.0182	08SD, 07HB
	AX-111806559	1A	449836347	1.83189E-05	0.0596	0.0243	07HB
	AX-110979338	1A	477467390	8.25251E-06	0.0655	0.0127	BLUP, 07HB, 09JS, 07SX, 09HB, 08SX, 08SD
	AX-111166388	1A	535329857	4.80243E-06	0.0631	0.0139	09JS, 08HB
	AX-109895974	1B	301047877	8.96464E-06	0.0647	0.0145	07SD, 09JS, 08SX
	AX-109980222	1B	410320741	1.19787E-05	0.0625	0.0195	BLUP, 08SC, 09JS, 08JS, 08SX, 07SX
	AX-108928321	1B	540990484	9.52681E-06	0.0644	0.0182	09SD, 08SD, 09JS, 08HB
	AX-111535339	1D	11159521	1.11302E-05	0.0631	0.0508	09SC
	AX-110627624	1D	40001986	2.24016E-06	0.0789	0.0060	BLUP, 08HB, 09JS, 08JS, 07JS, 09SD, 07SX, 09HB, 08SD, 08SX, 07HB, 08SC
	AX-111525866	1D	263206152	4.21568E-07	0.1028	0.0014	BLUP, 07HB, 08JS, 07JS, 08SD, 08SX, 09SD, 09SC, 09JS, 07SX, 08SC, 07SD, 09HB
	AX-110016930	1D	437447072	6.00689E-06	0.0667	0.0162	07SD
	AX-111946510	1D	478865288	3.79793E-06	0.0723	0.0080	BLUP, 08SX, 08SD, 07SX, 09HB
	AX-111584834	1D	482206429	8.46349E-07	0.1146	0.0019	BLUP, 07HB, 08HB, 09HB, 07SD, 08SD, 09SD, 08SC, 09SC, 07JS, 08JS, 09JS, 07SX, 08SX, 09SX
	AX-108842392	2A	216080379	2.47265E-06	0.0734	0.0058	09JS, 07SD, 08SX, 07HB
	AX-108891065	2A	247644605	7.14922E-07	0.1458	0.0072	BLUP, 08SD, 07SX, 08SX, 09JS, 09HB, 08JS, 07HB, 08HB, 07JS, 09SD, 07SD, 09SC, 08SC, 09SX
	AX-109344129	2A	717803941	1.45286E-05	0.0611	0.0261	07SD

AX-110563191	2B	129524648	1.60117E-05	0.0606	0.0263	07SD
AX-108913586	2B	439506437	3.23238E-06	0.0701	0.0063	08SD
AX-110412442	2B	651722410	2.72556E-06	0.0874	0.0063	BLUP, 08SX, 08SD, 09JS, 07SX, 08JS, 09HB, 07JS, 08SC, 07HB, 07SD, 08HB
AX-111551006	2B	706931127	3.6193E-06	0.0701	0.0072	BLUP, 08JS, 08SD, 09HB, 09JS, 07SX, 08SX
AX-111071301	2B	753951280	1.61783E-05	0.0610	0.0263	07SD
AX-108838533	2D	20349414	4.24079E-06	0.0729	0.0099	BLUP, 09JS, 07HB, 08JS, 09HB, 07SD, 07JS, 07SX, 08SD, 08SX, 09SD, 08HB, 08SC
AX-111772631	2D	20982836	4.78691E-06	0.0689	0.0111	BLUP, 09JS, 08SD, 07JS, 09SD, 09HB, 07SX, 08SX, 08HB, 08JS
AX-108885347	2D	78347091	1.75455E-05	0.0615	0.0173	08SD
AX-109508682	2D	247376233	5.24322E-06	0.0626	0.0140	09JS, 08HB
AX-94484517	2D	576287763	5.33787E-06	0.0676	0.0672	09SX
AX-110004274	2D	611223448	1.08112E-05	0.0639	0.0165	07HB, 08SD, 08JS
AX-111542571	2D	630384874	1.32961E-05	0.0619	0.0194	09JS, 09HB
AX-111039910	3A	23455186	4.08523E-06	0.0784	0.0079	BLUP, 08SD, 07HB, 07SX, 08JS, 09HB, 08SX
AX-111215306	3A	196496769	3.97965E-06	0.0676	0.0062	09JS
AX-109345729	3A	483161117	7.57802E-06	0.0681	0.0131	08SD, 08SX
AX-110192525	3A	511277955	1.53094E-05	0.0608	0.0263	07SD
AX-109060938	3A	537446832	1.5462E-05	0.0608	0.0174	09JS
AX-89381998	3A	637901043	7.12095E-06	0.0645	0.0224	08SC
AX-109870394	3A	673499714	3.37121E-06	0.0692	0.0089	07HB
AX-110589268	3A	729951582	1.42015E-05	0.0608	0.0261	07HB, 08HB
AX-111031240	3A	730657087	1.36517E-05	0.0623	0.0261	07SD
AX-110389997	3B	690013860	4.07679E-06	0.0684	0.0062	09JS
AX-109327802	4A	118749371	9.81419E-06	0.0644	0.0160	07HB, 08SD, 08JS
AX-109272207	4A	133050366	4.8286E-06	0.0567	0.0070	09JS
AX-110432225	4A	239579500	7.14337E-06	0.0664	0.0106	08SD
AX-111150736	4A	250043694	7.42786E-06	0.0651	0.0106	08SD
AX-109841603	4A	615169027	5.37498E-06	0.0705	0.0103	BLUP, 08SX, 07JS, 09HB
AX-111600193	4A	642368219	5.41604E-08	0.1750	0.0009	BLUP, 07HB, 08HB, 09HB, 07SD, 08SD, 09SD, 08SC, 09SC, 07JS, 08JS, 09JS, 07SX, 08SX, 09SX
AX-111031196	4B	149302606	1.04063E-05	0.0638	0.0165	BLUP, 09JS, 08SD, 08JS, 07SX
AX-111680609	4B	160309672	1.23488E-05	0.0622	0.0186	07HB, 08SD, 08JS
AX-111046402	4B	602873780	1.94588E-05	0.0591	0.0246	07HB
AX-169336707	4D	63	1.51429E-05	0.0605	0.0206	BLUP, 07HB
AX-109986929	4D	138	7.72359E-06	0.0662	0.0106	08SD
AX-169337600	4D	372	1.12276E-05	0.0637	0.0169	08SD, 08SX
AX-169338181	4D	433	1.59585E-05	0.0607	0.0171	08SD
AX-111020167	4D	471230915	1.14674E-05	0.0621	0.0232	08JS

AX-108769602	5A	483720326	1.97252E-05	0.0597	0.0310	07SD
AX-109893358	5A	480515122	6.93657E-06	0.0650	0.0134	07HB
AX-109897379	5A	454825148	3.45097E-06	0.0761	0.0085	BLUP, 09JS, 09HB, 07JS, 08JS, 07SX, 07HB, 08SC
AX-108844453	5A	453892447	2.96599E-06	0.0767	0.0097	BLUP, 09JS, 08SX, 09HB, 07HB, 07JS, 08JS, 09SD, 07SX, 08SD, 08SC, 09SC
AX-110015801	5A	391825899	9.29643E-06	0.0641	0.0120	09JS
AX-111043300	5B	296585755	7.42764E-06	0.0702	0.0122	08SD, 09JS, 09HB, 07HB
AX-111541781	5B	377348917	4.87009E-06	0.0646	0.0093	09JS, 08SX, 07SD, 07SX
AX-110433913	5B	490949803	1.28727E-05	0.0624	0.0261	07SD
AX-111495704	5B	645995677	1.83037E-05	0.0603	0.0243	07HB
AX-109025858	5B	654955111	7.01437E-06	0.0649	0.0252	08HB
AX-95152512	5D	14390791	1.12069E-06	0.1218	0.0034	BLUP, 08SD, 08SX, 07SX, 08JS, 09JS, 09HB, 07HB, 07JS, 09SD, 09SC, 07SD, 08SC, 08HB
AX-109963035	5D	237028854	1.02354E-05	0.0666	0.0156	BLUP, 07JS
AX-110616233	5D	434403329	1.07305E-05	0.0627	0.0170	07HB
AX-109809706	5D	476819411	9.33997E-06	0.0647	0.0161	BLUP, 08SX, 07SX, 08SD, 09SD, 09HB, 08JS
AX-108898503	5D	554823716	4.22984E-06	0.0682	0.0107	BLUP, 07SX, 09HB, 07HB, 08SC, 09SD
AX-86180093	5D	560329049	3.54393E-08	0.1654	0.0009	BLUP, 07HB, 08HB, 09HB, 07SD, 08SD, 09SD, 08SC, 09SC, 07JS, 08JS, 09JS, 07SX, 08SX, 09SX
AX-110946238	6A	18461050	2.72186E-06	0.0709	0.0057	08SD
AX-110012125	6A	614387818	1.07692E-05	0.0622	0.0170	07HB
AX-111686735	6B	144960242	7.92752E-06	0.0691	0.0144	08SX, 09JS, 08SD, 07SD, 09SD
AX-109317829	6B	236329093	4.03136E-06	0.0676	0.0062	09JS
AX-109084084	6B	567656373	8.56466E-07	0.1028	0.0040	BLUP, 08SX, 09HB, 08SD, 07SX, 09JS, 08JS, 07HB, 09SD, 07JS, 07SD, 08HB, 09SC
AX-110547035	6B	662869250	1.68081E-05	0.0600	0.1410	09SX
AX-94689627	6D	3917286	1.0667E-05	0.0636	0.0245	07SD
AX-109940039	6D	20554955	1.26073E-05	0.0618	0.0261	07SD
AX-109883908	6D	238655244	1.96375E-05	0.0599	0.0180	08SD
AX-111542213	7A	4553945	7.75608E-06	0.0651	0.0106	08SD
AX-89542612	7A	42500139	7.96349E-07	0.0782	0.0043	07SD
AX-110023271	7A	114087145	9.01634E-06	0.0639	0.0114	08SD
AX-109994561	7A	485512306	3.85891E-06	0.0693	0.0080	BLUP, 09JS, 07JS, 08JS, 07HB
AX-109397893	7A	673854089	7.23749E-06	0.0652	0.0170	09JS, 08HB
AX-111082615	7B	43512368	1.06147E-05	0.0531	0.0199	09JS, 07SD, 07JS
AX-94592534	7B	496271774	3.68902E-06	0.0713	0.0069	BLUP, 07SX, 09JS, 08SD, 08SX, 07SD
AX-110366783	7B	711490256	1.01088E-05	0.0644	0.0162	07JS, 08SC, 07HB, 09JS, 07SX, 08JS
AX-108911221	7D	4991311	9.97729E-07	0.0726	0.0040	BLUP, 09HB, 09JS, 08SX, 09SC, 07JS, 08JS, 08SD, 07SX, 07SD, 08SC, 09SD,

							09SX, 07HB
	AX-109943058	7D	14887102	8.49253E-06	0.0647	0.0113	09JS
	AX-111034889	7D	22777210	7.63352E-06	0.0651	0.0106	08SD
	AX-86172687	7D	23495477	1.24205E-06	0.0836	0.0034	BLUP, 09JS, 07JS, 08SC, 07SX, 08JS, 08HB, 07HB, 09HB, 08SX, 09SD
	AX-111197303	7D	47422389	5.79497E-06	0.0570	0.0093	BLUP, 09HB, 07SX, 08SD
	AX-110127261	7D	184864253	8.58013E-06	0.0643	0.0111	08SD
	AX-111218083	7D	191831492	1.08514E-05	0.0626	0.0171	08SX, 07SD, 09JS
	AX-109656174	7D	202543550	1.34045E-05	0.0623	0.0192	07HB, 08SD, 08JS
	AX-108948827	7D	265009497	3.06426E-06	0.0701	0.0062	08SD
	AX-94501611	7D	355562613	2.10744E-06	0.0724	0.0049	08SD
	AX-111503444	7D	393285287	1.41242E-06	0.0897	0.0038	BLUP, 08SX, 08SD, 09HB, 08JS, 09JS, 07JS, 07SD, 09SD, 07SX, 07HB, 08HB
	AX-110967909	7D	449077527	1.33549E-05	0.0639	0.0228	BLUP, 08SX, 07JS
	AX-109391680	7D	469085796	1.11516E-05	0.0532	0.0175	07HB, 08SD, 08JS
	AX-110826342	7D	480123355	5.01366E-06	0.0572	0.0084	08SD
	AX-108838800	7D	524655072	1.80084E-06	0.0879	0.0072	BLUP, 08SX, 08HB, 09JS, 08JS, 07SX, 08SD, 07SD, 07JS, 09SC
	AX-110362518	7D	529688251	5.08747E-06	0.0611	0.0142	09JS, 08HB
	AX-111505223	7D	544997456	1.91238E-05	0.0602	0.0180	08SD
	AX-111802901	7D	549281817	1.22859E-07	0.1144	0.0007	BLUP, 09HB, 09JS, 08SD, 08JS, 07HB, 07SX, 07JS, 08SX, 09SD, 07SD, 08SC, 08HB, 09SC
	AX-110446329	7D	554832615	3.00745E-06	0.0627	0.0066	BLUP, 08HB, 09JS, 08SX, 07JS, 08JS, 08SD, 09SD, 07SX, 09HB
	AX-108770812	7D	561926300	2.02598E-06	0.0725	0.0049	08SD
	AX-110016687	7D	563051214	4.55481E-07	0.1082	0.0013	BLUP, 08SD, 08HB, 07HB, 08SX, 09HB, 09JS, 07SX, 08JS, 09SC, 09SD, 07JS, 08SC, 07SD
	AX-94583542	7D	616037747	1.03586E-05	0.0639	0.0161	BLUP, 08SD, 07HB, 08JS
FSNS	AX-110979338	1A	477467390	1.40217E-05	0.0602	0.1007	09SD
	AX-111531574	1B	554497309	7.02812E-07	0.0776	0.0059	09HB
	AX-108758276	1B	647870029	2.77232E-06	0.0710	0.0378	07SD
	AX-111525866	1D	263206152	2.79464E-06	0.0749	0.0208	09HB, 09JS
	AX-108891065	2A	247644605	5.77319E-06	0.0685	0.0343	BLUP, 08JS, 09JS, 07JS
	AX-109599637	2A	315321960	1.93175E-05	0.0628	0.0648	07JS
	AX-110529731	2A	734391881	1.60697E-05	0.0599	0.1083	07HB
	AX-110412442	2B	651722410	2.96355E-06	0.0708	0.0332	BLUP, 09SD
	AX-89577616	3D	355222696	1.55467E-05	0.0508	0.0648	07JS
	AX-111600193	4A	642368219	3.6542E-07	0.1041	0.0075	BLUP, 07JS, 08JS, 09JS, 09SD, 07HB, 09HB, 08SC, 08HB, 09SC, 07SD
	AX-110963243	4B	171658915	1.72338E-05	0.0641	0.1083	07HB
	AX-111620283	4D	101445917	1.22521E-05	0.0639	0.1033	07HB
	AX-110036471	5A	449037663	4.68903E-07	0.0837	0.0036	BLUP, 08JS, 07JS

	AX-111043300	5B	296585755	1.42635E-06	0.0770	0.0104	BLUP, 07JS, 08JS
	AX-109860828	5B	422030835	1.25493E-06	0.0747	0.0079	09HB
	AX-108891984	5B	565531627	1.73494E-05	0.0598	0.0648	07JS
	AX-95152512	5D	14390791	1.63556E-06	0.0928	0.0157	BLUP, 07HB, 08HB, 09HB, 07JS, 08JS, 09JS, 08SC, 07SD
	AX-94567087	5D	478739552	9.82711E-06	0.0659	0.1651	09SC
	AX-89675057	5D	538012085	1.44526E-05	0.0613	0.0648	07JS
	AX-109274468	5D	543650147	1.57985E-06	0.0744	0.0378	07SD
	AX-94413800	5D	547527234	5.86471E-06	0.0667	0.0492	07SD
	AX-86180093	5D	560329049	1.87908E-06	0.1053	0.0240	BLUP, 07JS, 08JS, 09JS, 08SC, 09HB, 09SD, 07HB, 07SD, 09SC
	AX-111554374	6A	84033013	8.42719E-06	0.0668	0.0323	BLUP, 09HB, 07JS
	AX-108831922	6A	558112855	7.49494E-06	0.0657	0.0308	09HB
	AX-110433950	6B	171767664	5.36378E-06	0.0669	0.0338	07JS
	AX-109084084	6B	567656373	3.67453E-06	0.0821	0.0309	BLUP, 09JS, 08JS, 07JS, 09SD, 07HB
	AX-109476705	6B	614603892	0.0000144	0.0611	0.0558	BLUP
	AX-89324800	6D	4371990	5.05446E-06	0.0674	0.0492	07SD
	AX-109528482	6D	65642547	5.17593E-06	0.0653	0.0593	09SD
	AX-108918745	6D	427484647	3.00037E-06	0.0715	0.0378	07SD
	AX-111473122	7A	16653198	1.10004E-05	0.0626	0.0554	07JS
	AX-110023271	7A	114087145	3.42641E-06	0.0871	0.0346	BLUP, 07JS, 08JS, 09JS, 09HB, 09SD, 08SC
	AX-109994561	7A	485512306	5.44985E-06	0.0664	0.0250	09HB
	AX-110020351	7B	327102495	3.62311E-06	0.0581	0.0456	07HB
	AX-109822071	7B	524601841	5.14649E-06	0.0677	0.0250	09HB
	AX-109429982	7B	660663186	2.45955E-06	0.0723	0.0138	09HB
	AX-110503493	7D	40499462	4.54152E-06	0.0713	0.0181	09JS, 09HB
	AX-111503444	7D	393285287	8.88556E-06	0.0646	0.0971	BLUP, 08JS, 08SC, 08HB
	AX-110349900	7D	528073469	1.31096E-05	0.0618	0.0643	09HB, 09JS
	AX-109742925	7D	548906076	1.58443E-05	0.0603	0.0796	08JS
	AX-111802901	7D	549281817	0.00000525	0.0662	0.0240	BLUP
SNPP	AX-110563049	1A	15905547	0.0000122	0.0615	0.0077	BLUP
	AX-110375459	1A	55931109	0.0000172	0.0587	0.0077	BLUP
	AX-109841934	1A	74429581	0.00000451	0.0660	0.0060	BLUP
	AX-111736507	1A	101770682	0.00000524	0.0657	0.0060	BLUP
	AX-111681662	1A	480954488	0.0000121	0.0510	0.0077	BLUP
	AX-110597912	1A	537536897	0.00000644	0.0645	0.0065	BLUP
	AX-108770381	1A	547718158	1.68233E-06	0.0717	0.0809	08JS
	AX-110480800	1A	548491178	1.88214E-05	0.0585	0.0395	08SX
	AX-110595289	1A	582835553	0.0000168	0.0493	0.0077	BLUP
	AX-109895974	1B	301047877	1.66149E-05	0.0591	0.0836	08HB
	AX-109876087	1B	373317133	7.63474E-06	0.0661	0.0170	BLUP, 08SX, 09SX, 07SX, 08SD
	AX-109302867	1B	383054878	1.07455E-05	0.0617	0.0770	08HB
	AX-109347269	1B	409702951	0.00000212	0.0703	0.0046	BLUP

AX-109032696	1B	442501925	8.7638E-06	0.0627	0.0770	08HB
AX-111087865	1B	542639626	0.00000461	0.0661	0.0060	BLUP
AX-109951074	1B	681503047	0.00000912	0.0623	0.0073	BLUP
AX-111561055	1D	13796245	0.0000172	0.0494	0.0077	BLUP
AX-110296535	1D	21640510	0.0000166	0.0588	0.0077	BLUP
AX-108883328	1D	30879589	0.00000316	0.0684	0.0053	BLUP
AX-109887148	1D	38521795	0.0000175	0.0586	0.0077	BLUP
AX-110434289	1D	46215179	1.95732E-05	0.0587	0.0898	08HB
AX-111525866	1D	263206152	4.65581E-08	0.0930	0.0005	09JS
AX-111066559	1D	278099919	3.4616E-06	0.0673	0.1743	07HB
AX-108842392	2A	216080379	1.49145E-05	0.0597	0.0836	08HB
AX-108891065	2A	247644605	1.07453E-07	0.0878	0.0008	09JS
AX-111263159	2A	256849714	0.00000699	0.0636	0.0065	BLUP
AX-110921503	2B	8351159	0.0000185	0.0582	0.0078	BLUP
AX-108846215	2B	139386906	0.00000936	0.0629	0.0073	BLUP
AX-110974562	2B	190657526	0.000018	0.0583	0.0077	BLUP
AX-108838753	2B	244212095	0.00000699	0.0640	0.0065	BLUP
AX-109517040	2B	596918194	0.000000941	0.0755	0.0028	BLUP
AX-109919527	2B	622066161	8.98669E-06	0.0636	0.0906	08JS
AX-111762792	2B	785093834	0.0000177	0.0584	0.0077	BLUP
AX-111531844	2B	795488728	0.0000159	0.0591	0.0077	BLUP
AX-108838533	2D	20349414	5.4284E-06	0.0655	0.0157	BLUP, 08SX, 07SX, 09SX, 08SD
AX-111772631	2D	20982836	9.24245E-08	0.0893	0.0008	09JS
AX-110480398	2D	60951241	0.0000116	0.0513	0.0077	BLUP
AX-111304532	2D	63742084	0.0000116	0.0512	0.0077	BLUP
AX-109503554	2D	71459296	1.21438E-05	0.0614	0.0290	07SX
AX-110391203	2D	304264498	0.00000446	0.0661	0.0060	BLUP
AX-111016259	2D	571001937	0.00000252	0.0693	0.0051	BLUP
AX-110574174	2D	607416371	0.0000175	0.0585	0.0077	BLUP
AX-111611367	3A	36657625	0.0000124	0.0509	0.0077	BLUP
AX-108972085	3A	37823933	0.00000985	0.0617	0.0074	BLUP
AX-110570804	3A	69061323	0.00000358	0.0674	0.0058	BLUP
AX-108938752	3A	524768985	0.00000893	0.0628	0.0073	BLUP
AX-109474344	3A	544461911	1.52402E-06	0.0724	0.0191	08HB
AX-109851882	3A	653326855	0.00000776	0.0632	0.0067	BLUP
AX-109612604	3A	663669493	0.00000701	0.0638	0.0065	BLUP
AX-110020522	3A	686168545	2.43157E-07	0.0830	0.0012	BLUP, 07SX, 08SX, 09SX, 08SD
AX-111701890	3A	718808256	0.0000174	0.0586	0.0077	BLUP
AX-109110573	3B	240483400	0.00000806	0.0629	0.0069	BLUP
AX-111564046	3B	419278534	0.00000532	0.0652	0.0060	BLUP
AX-111474667	3B	477364176	0.00000424	0.0663	0.0059	BLUP
AX-109365701	3B	512507145	1.38074E-05	0.0618	0.1347	07SD
AX-111286422	3B	572601237	0.00000412	0.0666	0.0059	BLUP
AX-110142073	3B	661319698	1.95209E-07	0.0845	0.0011	BLUP, 07SX, 08SX, 09SX, 08SD

AX-109396253	3B	762092326	7.52252E-06	0.0650	0.0699	BLUP, 07SD
AX-108837323	3B	780329971	1.9613E-05	0.0578	0.0429	07SX
AX-109981452	3D	6728754	5.76359E-06	0.0643	0.0809	08JS
AX-111080889	3D	47497225	0.000017	0.0587	0.0077	BLUP
AX-109179417	3D	128789720	0.00000305	0.0682	0.0053	BLUP
AX-109471652	3D	268152441	0.00000585	0.0648	0.0061	BLUP
AX-108796887	3D	309005096	0.0000102	0.0519	0.0075	BLUP
AX-108765756	3D	604157383	0.00000199	0.0801	0.0046	BLUP
AX-109627140	4A	122281521	0.000018	0.0585	0.0077	BLUP
AX-109066809	4A	179941571	0.000000606	0.0822	0.0023	BLUP
AX-109465075	4A	206684337	0.0000165	0.0589	0.0077	BLUP
AX-111600193	4A	642368219	8.34307E-07	0.2087	0.0128	BLUP, 07SX, 08SX, 08SD, 09SX, 09JS, 09SD, 08JS
AX-109845365	4A	706599745	0.0000169	0.0593	0.0077	BLUP
AX-109901718	4A	715743670	0.0000127	0.0605	0.0077	BLUP
AX-110925399	4B	51858889	0.00000605	0.0644	0.0062	BLUP
AX-111031196	4B	149302606	0.0000121	0.0621	0.0077	BLUP
AX-109400188	4B	459007802	0.00000507	0.0653	0.0060	BLUP
AX-110442771	4B	466640145	0.0000169	0.0587	0.0077	BLUP
AX-108941085	4B	608942788	0.0000196	0.0580	0.0081	BLUP
AX-110445790	4B	646452051	2.35759E-06	0.0694	0.0085	07SX
AX-169337207	4D	258	2.12783E-06	0.0742	0.0066	BLUP, 09HB
AX-110033504	5A	21418196	8.92645E-12	0.1447	0.0000	BLUP, 07SX, 08SX, 09SX, 08SD
AX-109884758	5A	463520339	2.20466E-06	0.0605	0.0079	08SX
AX-108741695	5A	519880017	0.0000143	0.0597	0.0077	BLUP
AX-109952547	5A	568087699	0.00000743	0.0633	0.0066	BLUP
AX-111051748	5B	44358277	6.7909E-07	0.0667	0.0029	08SX
AX-111723779	5B	142666541	0.0000107	0.0617	0.0076	BLUP
AX-111043300	5B	296585755	5.09216E-06	0.0665	0.0214	09JS
AX-110576186	5B	398060370	0.0000155	0.0602	0.0077	BLUP
AX-111556817	5B	404142346	0.0000144	0.0596	0.0077	BLUP
AX-111552581	5B	448128619	0.00000164	0.0728	0.0039	BLUP
AX-110924307	5B	514686625	1.06114E-05	0.0624	0.1347	07SD
AX-110369532	5B	566494041	1.03792E-06	0.0741	0.0191	08HB
AX-110586945	5B	584692291	2.18909E-06	0.0785	0.0081	BLUP, 07SX, 08SX, 09SX, 08SD
AX-95152512	5D	14390791	2.20231E-06	0.1796	0.0329	BLUP, 07SX, 08SX, 09SX, 08SD, 09JS
AX-110379095	5D	193521326	0.0000148	0.0594	0.0077	BLUP
AX-108986002	5D	213131917	0.0000156	0.0601	0.0077	BLUP
AX-89349755	5D	250056711	0.00000501	0.0654	0.0060	BLUP
AX-111607088	5D	382077176	0.00000976	0.0617	0.0074	BLUP
AX-108865212	5D	415746946	0.0000147	0.0595	0.0077	BLUP
AX-95203327	5D	462083496	0.0000172	0.0586	0.0077	BLUP
AX-86180093	5D	560329049	5.87572E-07	0.1915	0.0148	BLUP, 07SX, 08SX, 08SD, 09SX, 09JS, 09SD

AX-111259000	6A	7054819	0.0000176	0.0585	0.0077	BLUP
AX-111233375	6A	18560088	0.00000424	0.0675	0.0059	BLUP
AX-110720861	6A	25535895	7.37831E-06	0.0644	0.0183	BLUP, 07SX, 08SX, 09SX, 08SD
AX-109904205	6A	228192127	1.2427E-05	0.0610	0.0300	08SX
AX-111565588	6A	270300881	0.0000141	0.0597	0.0077	BLUP
AX-111558975	6A	415196444	5.33835E-06	0.0660	0.0138	BLUP, 07SX, 08SX, 09SX, 08SD
AX-108908139	6B	99681079	0.000000815	0.0772	0.0027	BLUP
AX-111686735	6B	144960242	6.78395E-07	0.0766	0.0191	08HB
AX-111082654	6B	343970282	0.0000134	0.0600	0.0077	BLUP
AX-111623534	6B	433157274	0.0000166	0.0596	0.0077	BLUP
AX-109983347	6B	461592292	0.000017	0.0587	0.0077	BLUP
AX-109084084	6B	567656373	6.06897E-08	0.1261	0.0003	BLUP, 07SX, 09SX, 08SX, 08SD, 09JS
AX-108965440	6B	571046841	0.000017	0.0588	0.0077	BLUP
AX-110559317	6B	590758257	0.00000718	0.0639	0.0065	BLUP
AX-110687567	6B	707208262	0.0000128	0.0604	0.0077	BLUP
AX-94546836	6D	4410702	1.82149E-05	0.0492	0.0395	08SX
AX-94941823	6D	5987200	0.0000156	0.0591	0.0077	BLUP
AX-109399289	6D	6831687	0.0000171	0.0588	0.0077	BLUP
AX-111706410	6D	7104958	0.0000104	0.0615	0.0075	BLUP
AX-109897923	6D	7291908	0.0000141	0.0597	0.0077	BLUP
AX-111913895	6D	47060228	0.0000189	0.0581	0.0079	BLUP
AX-109088524	6D	383981282	6.28111E-06	0.0650	0.0115	BLUP, 07SX
AX-95256889	6D	400546291	0.00000374	0.0670	0.0059	BLUP
AX-111696257	6D	431111546	0.0000173	0.0586	0.0077	BLUP
AX-110190299	6D	472984300	0.0000173	0.0586	0.0077	BLUP
AX-110023271	7A	114087145	1.63727E-06	0.0724	0.0075	09JS
AX-111064790	7A	281664921	6.30568E-06	0.0675	0.0167	BLUP, 07SX, 08SX
AX-110394064	7A	284611580	0.0000143	0.0597	0.0077	BLUP
AX-110382669	7A	492086154	0.00000249	0.0702	0.0051	BLUP
AX-108763286	7A	528403659	7.16245E-06	0.0644	0.0703	BLUP, 07SD
AX-110401938	7A	610205797	0.0000181	0.0584	0.0077	BLUP
AX-110412676	7A	701813329	0.0000149	0.0596	0.0077	BLUP
AX-110511347	7A	704290704	0.00000914	0.0628	0.0073	BLUP
AX-111147188	7B	36333209	0.00000557	0.0648	0.0061	BLUP
AX-111181637	7B	48481023	1.75967E-05	0.0494	0.0395	08SX
AX-108776567	7B	57935751	0.000000751	0.0769	0.0027	BLUP
AX-109324588	7B	344080087	0.0000104	0.0620	0.0075	BLUP
AX-109306202	7B	568676196	1.95613E-07	0.0843	0.0012	BLUP, 07SX, 08SX, 09SX, 08SD
AX-109347474	7B	609141487	0.0000138	0.0613	0.0077	BLUP
AX-89703681	7B	639791862	5.61948E-06	0.0718	0.0177	BLUP, 07SX, 08SD
AX-109974767	7B	688599354	4.66145E-07	0.0809	0.0196	BLUP, 07SD
AX-108865688	7B	690239330	0.00000585	0.0648	0.0061	BLUP
AX-86178207	7B	706808987	1.49645E-06	0.0620	0.0191	08HB
AX-95684576	7B	706861473	0.000000926	0.0755	0.0028	BLUP

	AX-110476719	7B	712889242	0.00000264	0.0693	0.0051	BLUP
	AX-108746141	7B	718332571	0.0000093	0.0632	0.0073	BLUP
	AX-110581635	7B	720497627	1.54298E-05	0.0497	0.0836	08HB
	AX-110595065	7B	723154830	0.0000111	0.0613	0.0077	BLUP
	AX-108911221	7D	4991311	5.73739E-09	0.0969	0.0001	09JS
	AX-110541194	7D	337568893	0.0000029	0.0685	0.0052	BLUP
	AX-111503444	7D	393285287	5.71225E-06	0.0680	0.0419	09JS, 08HB
	AX-110913995	7D	428054733	1.47942E-05	0.0596	0.0309	BLUP, 07SX, 08SX, 09SX
	AX-110967909	7D	449077527	5.70758E-08	0.0987	0.0004	BLUP, 07SX, 08SX, 09SX, 08SD
	AX-108838800	7D	524655072	1.53183E-05	0.0602	0.0551	09JS
	AX-111802901	7D	549281817	2.56081E-08	0.1374	0.0002	BLUP, 07SX, 08SX, 09SX, 08SD, 09JS
	AX-110446329	7D	554832615	9.08021E-07	0.0653	0.0035	BLUP, 07SX, 08SX, 09SX, 08SD
	AX-110016687	7D	563051214	8.28845E-06	0.0636	0.0321	09JS
	AX-109835957	7D	632128283	6.41522E-06	0.0664	0.0809	08JS
PH	AX-108800518	1A	54587011	9.76705E-07	0.0765	0.0123	07SD
	AX-108912867	1A	474384177	2.23931E-06	0.0715	0.0170	07SD
	AX-111531574	1B	554497309	5.78625E-06	0.0698	0.0275	BLUP, 09HB, 07SD
	AX-111525866	1D	263206152	5.50955E-06	0.0686	0.0346	BLUP, 07SX, 08SX, 09SX, 07SC
	AX-89612565	1D	320382523	1.81359E-05	0.0589	0.0852	08SX
	AX-111667781	2B	50354330	2.34858E-06	0.0726	0.0198	08SC
	AX-110452530	2B	433964309	1.19616E-05	0.0617	0.0465	07SD
	AX-111132985	2D	8670932	1.30192E-06	0.0775	0.0113	BLUP, 08SC
	AX-110621158	2D	16015354	8.55415E-08	0.0911	0.0014	08SC
	AX-111498546	3A	624694401	5.83486E-06	0.0742	0.0466	BLUP, 07SX, 07JS, 07HB
	AX-111577792	3A	714283618	1.01053E-05	0.0630	0.0849	09SX, 09JS
	AX-110177992	3A	723214155	6.66567E-06	0.0650	0.0517	BLUP, 07HB, 07SX, 07JS
	AX-109401854	3B	80092394	3.92364E-06	0.0682	0.0219	07SD
	AX-109456365	3B	325678290	1.42689E-05	0.0607	0.1441	09SC
	AX-109471812	3B	827132879	2.31018E-07	0.0842	0.0058	07SD
	AX-109272207	4A	133050366	1.07275E-05	0.0522	0.0711	BLUP, 07JS, 07HB
	AX-109973724	4A	247148243	8.70009E-06	0.0527	0.0548	08SX
	AX-111600193	4A	642368219	8.63821E-07	0.0850	0.0325	BLUP, 07HB, 09HB, 07SD, 08SD, 09SD, 07SC, 08SC, 09SC, 07JS, 08JS, 09JS, 07SX, 08SX, 09SX
	AX-108745433	4A	703576045	1.25184E-05	0.0609	0.0800	BLUP, 08SX, 09HB
	AX-111651544	4B	427276284	1.90933E-05	0.0590	0.0802	08SC
	AX-109404379	4B	546372605	1.17671E-05	0.0624	0.0465	07SD
	AX-169336388	4D	19	1.74561E-05	0.0594	0.0802	08SC
	AX-108890219	4D	486	5.85476E-06	0.0677	0.0740	09JS, 08SC, 07SX, 09SC, 08SD
	AX-109940654	5A	568661654	4.58053E-06	0.0692	0.0231	07SD
	AX-109860828	5B	422030835	3.24932E-06	0.0748	0.0422	BLUP, 07HB, 08HB, 09HB, 07SD, 07SX, 08SX, 09SX, 07JS, 08JS
	AX-95152512	5D	14390791	4.43595E-06	0.0744	0.0395	BLUP, 09HB, 08SX, 09SX, 09SD, 07SD, 08SC, 07HB, 07SX, 07JS, 08JS

AX-109809706	5D	476819411	1.15292E-05	0.0631	0.1159	09JS
AX-108898503	5D	554823716	7.32574E-06	0.0652	0.0923	09JS
AX-86180093	5D	560329049	1.76403E-05	0.0593	0.0802	08SC
AX-111554374	6A	84033013	2.78666E-06	0.0753	0.0370	BLUP, 07HB, 07SD, 07SX, 09SX, 08HB, 09HB, 08JS
AX-109337275	6B	400888529	5.12739E-06	0.0709	0.0294	08SC
AX-109084084	6B	567656373	1.98554E-06	0.0759	0.0167	08SX
AX-110367735	6D	68132618	1.69209E-05	0.0599	0.0608	07SD
AX-110038044	7A	484716540	1.8552E-06	0.0733	0.0312	09JS
AX-109469020	7A	637965507	3.22406E-06	0.0716	0.0232	08SC
AX-109429982	7B	660663186	5.17576E-06	0.0733	0.0515	BLUP, 07HB, 09HB, 08SX, 07SD, 07SX, 08HB, 09SX, 07JS, 08SD
AX-111568844	7B	505447589	6.36228E-06	0.0788	0.0258	BLUP, 08SC
AX-111058442	7D	58203663	7.19824E-06	0.0645	0.1323	09SC
AX-108748467	7D	263881844	1.86179E-05	0.0596	0.0852	08SX
AX-109526545	7D	397577441	1.02405E-05	0.0628	0.0577	BLUP, 07HB, 07SX, 07JS
AX-109391680	7D	469085796	1.85023E-05	0.0487	0.0852	08SX
AX-111157930	7D	513389248	1.97034E-05	0.0590	0.0662	07SD
AX-110396689	7D	610620986	1.19936E-05	0.0631	0.1441	09SC

SL, spike length; TKW, 1000-kernel weight; KNS, kernel number per spike; TSNS, total spikelet number per spike; FSNS, fertile spikelet number per spike; GWS, grain weight per spike; PH, plant height; SNPP, spike number per plant; SX, HB, SD, SC and JS refer to Shaanxi, Hebei, Shandong, Sichuan and Jiangsu locations, respectively. 07, 08 and 09 refer to 2007, 2008 and 2009 year, respectively

TABLE S6 Distribution of the significant SNPs for eight agronomic traits on 21 wheat chromosomes.

Chr	PH	SL	TSNS	FSNS	KNS	TKW	SNPP	GWS
1A	2	5	4	1	2		9	2
1B	1	5	3	2	2		7	2
1D	2	5	6	1	2	1	7	4
2A		3	3	3	2	1	3	5
2B	2	5	5	1	2		8	2
2D	2	5	7		4		8	2
3A	3	6	9		1		9	4
3B	3	3	1		2		8	6
3D		4		1		1	6	
4A	4	6	6	1		1	6	4
4B	2	4	3	1	1		6	4
4D	2	3	5	1		1	1	2
5A	1	4	5	1	2		4	4
5B	1	6	5	3		1	9	7
5D	4	6	6	6	1	1	8	5
6A	1	4	2	2	4	1	6	3
6B	2	4	4	3	5		9	3

6D	1	1	3	3			10	3
7A	2	6	5	3	2	1	8	6
7B	2	4	3	3		1	15	4
7D	6	20	22	5	2	3	10	9
Total	43	109	107	41	34	13	157	81

SL, spike length; TKW, 1000-kernel weight; KNS, kernel number per spike; TSNS, total spikelet number per spike; FSNS, fertile spikelet number per spike; GWS, grain weight per spike; PH, plant height; SNPP, spike number per plant

TABLE S7 Comparison of phenotypes in 15 environments between cultivars containing superior alleles and cultivars containing inferior alleles at 31 loci. Superior alleles are showed in red.

Trait	Chr	SNP	Allele	2007					2008					2009				
				Shaanxi	Jiangsu	Hebei	Shandong	Sichuan	Shaanxi	Jiangsu	Hebei	Shandong	Sichuan	Shaanxi	Jiangsu	Hebei	Shandong	Sichuan
TKW	4A	AX-111600193	A/A	38.589	39.756	40.545	36.251	39.624	42.518	37.775	37.866	38.289	40.490	38.942	37.102	35.972	39.883	39.855
			G/G	36.628	38.186	39.168	34.331	37.500	40.471	36.166	36.179	35.172	38.722	38.104	34.804	34.439	39.428	35.420
			P	0.003	0.036	0.054	0.012	0.019	0.003	0.033	0.019	0.001	0.010	0.112	0.001	0.012	0.271	0.000
	5B	AX-109860828	A/A	38.378	39.862	40.576	36.107	39.732	42.580	37.611	37.648	38.197	40.678	38.791	37.153	35.985	39.750	39.779
			G/G	37.546	37.954	39.417	35.544	37.011	40.817	36.868	36.863	36.248	37.910	38.597	35.136	34.513	39.641	36.814
			P	0.082	0.004	0.051	0.205	0.001	0.002	0.155	0.125	0.007	0.000	0.366	0.000	0.005	0.430	0.001
	7D	AX-108838800	A/A	38.271	39.786	40.383	36.201	39.909	42.395	37.882	37.703	38.328	40.537	38.739	37.019	35.824	39.840	39.943
			C/C	37.433	37.664	39.270	35.453	35.875	40.684	35.144	36.276	35.759	37.998	38.230	34.972	34.616	39.498	35.509
			P	0.078	0.002	0.057	0.142	0.000	0.003	0.000	0.018	0.001	0.000	0.186	0.000	0.015	0.293	0.000
KNS	2D	AX-110982403	G/G	50.938	49.507	54.650	46.007	44.147	50.300	49.185	48.653	39.718	31.510	50.518	47.585	51.359	41.166	33.319
			T/T	53.278	55.361	59.931	50.979	49.257	53.051	55.195	53.000	42.873	35.282	52.658	54.816	55.669	43.941	36.515
			P	0.001	0.000	0.000	0.000	0.000	0.000	0.000	0.000	0.000	0.000	0.004	0.000	0.000	0.000	0.000
TSNS	1B	AX-108928321	A/A	19.546	22.480	21.160	19.208	—	20.242	21.671	19.674	18.672	21.087	15.940	21.375	19.745	19.426	21.471
			G/G	18.795	21.614	20.473	18.489	—	19.430	20.805	19.371	18.370	20.322	15.583	20.798	19.197	18.873	20.961
			P	0.000	0.000	0.000	0.000	—	0.000	0.000	0.078	0.004	0.000	0.100	0.000	0.000	0.000	0.001
	1D	AX-110627624	C/C	19.005	21.643	20.535	18.644	—	19.488	21.001	19.433	18.270	20.524	15.629	20.790	19.314	18.850	21.079
			T/T	19.281	22.625	21.254	19.073	—	20.251	21.525	19.591	18.912	20.862	15.919	21.529	19.589	19.471	21.326
			P	0.056	0.000	0.000	0.011	—	0.000	0.001	0.233	0.000	0.026	0.157	0.000	0.049	0.000	0.087
	2D	AX-109508682	A/A	18.793	21.576	20.360	18.425	—	19.434	20.938	19.042	18.338	20.455	15.122	20.734	19.192	18.615	21.113
			G/G	19.221	22.103	20.915	18.975	—	19.900	21.205	19.675	18.536	20.680	15.906	21.137	19.472	19.270	21.151
			P	0.007	0.002	0.005	0.002	—	0.008	0.063	0.002	0.052	0.099	0.003	0.005	0.047	0.000	0.419
	3A	AX-110589268	A/A	19.062	21.789	20.762	18.575	—	19.563	21.048	19.211	18.396	20.546	15.372	20.916	19.267	18.981	21.042
			C/C	19.193	22.158	20.801	19.056	—	20.004	21.346	19.774	18.661	20.759	16.320	21.149	19.658	19.268	21.375
			P	0.221	0.024	0.426	0.005	—	0.009	0.042	0.004	0.012	0.105	0.000	0.069	0.009	0.030	0.025

	4D	AX-169337600	A/A	18.940	21.569	20.425	18.494	—	19.381	21.004	19.183	18.227	20.622	15.089	20.651	19.310	18.686	21.090
			G/G	19.207	22.163	20.934	18.916	—	19.980	21.217	19.619	18.640	20.645	15.935	21.218	19.464	19.327	21.182
			P	0.069	0.001	0.009	0.014	—	0.001	0.122	0.028	0.000	0.449	0.002	0.000	0.189	0.000	0.312
	5A	AX-108844453	A/A	19.354	22.333	21.006	19.042	—	20.175	21.283	19.668	18.617	20.754	15.992	21.248	19.523	19.404	21.310
			C/C	18.878	21.619	20.557	18.487	—	19.324	21.072	19.284	18.361	20.515	15.505	20.817	19.346	18.800	21.018
			P	0.002	0.000	0.014	0.001	—	0.000	0.105	0.034	0.013	0.076	0.037	0.003	0.136	0.000	0.040
	5B	AX-111541781	A/A	19.446	22.364	21.027	19.018	—	20.124	21.418	19.579	18.618	20.885	15.734	21.305	19.512	19.313	21.311
			G/G	18.810	21.596	20.500	18.522	—	19.441	20.944	19.336	18.383	20.385	15.690	20.784	19.283	18.890	20.989
			P	0.000	0.000	0.005	0.003	—	0.000	0.003	0.131	0.021	0.001	0.438	0.000	0.079	0.003	0.033
	7B	AX-111082615	A/A	19.241	22.119	21.044	18.782	—	19.783	21.414	19.798	18.592	20.889	15.629	21.187	19.598	19.119	21.313
			T/T	18.910	21.779	20.437	18.757	—	19.776	20.877	19.096	18.406	20.316	15.807	20.854	19.186	19.071	20.947
			P	0.023	0.030	0.001	0.445	—	0.484	0.001	0.000	0.053	0.000	0.256	0.015	0.005	0.375	0.017
	7D	AX-110967909	C/C	18.795	21.577	20.581	18.422	—	19.157	21.015	19.253	18.366	20.407	15.546	20.808	19.379	18.794	21.008
			T/T	19.324	22.261	20.935	19.030	—	20.142	21.302	19.617	18.583	20.818	15.813	21.226	19.480	19.336	21.242
			P	0.001	0.000	0.045	0.000	—	0.000	0.047	0.048	0.036	0.007	0.175	0.004	0.271	0.000	0.090
GWS	3B	AX-110142073	C/C	2.007	2.298	2.374	1.767	1.904	2.217	2.347	1.931	1.553	1.377	2.059	1.896	1.956	1.719	1.417
			G/G	1.936	2.258	2.191	1.674	1.746	2.098	2.235	1.828	1.537	1.268	1.904	1.816	1.809	1.639	1.279
			P	0.023	0.225	0.001	0.013	0.009	0.000	0.011	0.008	0.329	0.000	0.000	0.037	0.000	0.004	0.000
	4A	AX-110630537	C/C	1.929	2.209	2.184	1.670	1.763	2.092	2.224	1.820	1.504	1.267	1.933	1.795	1.819	1.637	1.274
			T/T	2.091	2.475	2.545	1.865	1.998	2.344	2.497	2.059	1.677	1.497	2.131	1.987	2.049	1.806	1.554
			P	0.000	0.000	0.000	0.000	0.001	0.000	0.000	0.000	0.000	0.000	0.000	0.000	0.000	0.000	0.000
	4A	AX-111600193	A/A	2.009	2.297	2.324	1.764	1.849	2.190	2.313	1.917	1.586	1.357	2.009	1.901	1.909	1.698	1.386
			G/G	1.850	2.250	2.141	1.573	1.750	2.066	2.281	1.787	1.397	1.249	1.944	1.664	1.791	1.630	1.215
			P	0.000	0.258	0.010	0.001	0.143	0.006	0.322	0.014	0.000	0.007	0.111	0.000	0.012	0.053	0.001
	5A	AX-110913131	C/C	1.892	2.162	2.127	1.607	1.674	2.058	2.143	1.741	1.458	1.204	1.905	1.691	1.796	1.614	1.242
			T/T	2.031	2.356	2.386	1.813	1.923	2.226	2.391	1.978	1.602	1.403	2.035	1.972	1.943	1.726	1.422
			P	0.000	0.000	0.000	0.000	0.000	0.000	0.000	0.000	0.000	0.000	0.000	0.000	0.000	0.000	0.000
	5A	AX-110033504	C/C	2.033	2.403	2.393	1.799	1.932	2.260	2.420	2.012	1.639	1.420	2.048	1.966	1.979	1.747	1.465
			T/T	1.918	2.168	2.181	1.662	1.713	2.070	2.190	1.781	1.471	1.244	1.930	1.755	1.803	1.621	1.248

			P	0.000	0.000	0.000	0.000	0.000	0.000	0.000	0.000	0.000	0.000	0.001	0.000	0.000	0.000	0.000
	5B	AX-110586945	C/C	2.030	2.387	2.458	1.770	1.902	2.275	2.452	1.961	1.626	1.421	2.061	1.912	1.989	1.740	1.457
			T/T	1.937	2.208	2.169	1.693	1.756	2.089	2.195	1.841	1.503	1.270	1.937	1.803	1.813	1.639	1.275
			P	0.005	0.000	0.000	0.036	0.016	0.000	0.000	0.003	0.000	0.000	0.001	0.009	0.000	0.001	0.000
	5D	AX-95152512	C/C	1.985	2.308	2.292	1.750	1.837	2.176	2.311	1.900	1.560	1.337	2.000	1.878	1.899	1.694	1.362
			G/G	1.886	2.042	2.193	1.532	1.605	2.031	2.120	1.719	1.456	1.245	1.874	1.574	1.710	1.518	1.167
			P	0.054	0.002	0.157	0.001	0.024	0.009	0.013	0.008	0.049	0.052	0.029	0.000	0.002	0.000	0.001
	6B	AX-109084084	A/A	1.854	2.046	2.118	1.624	1.661	1.988	2.096	1.673	1.408	1.167	1.881	1.617	1.742	1.561	1.182
			G/G	2.006	2.347	2.328	1.752	1.869	2.206	2.350	1.938	1.585	1.373	2.013	1.914	1.916	1.712	1.400
			P	0.000	0.000	0.001	0.005	0.004	0.000	0.000	0.000	0.000	0.000	0.002	0.000	0.000	0.000	0.000
	6D	AX-95009966	C/C	2.011	2.313	2.334	1.743	1.890	2.209	2.313	1.890	1.568	1.354	2.027	1.919	1.918	1.712	1.388
			T/T	1.931	2.248	2.232	1.698	1.745	2.102	2.287	1.870	1.523	1.287	1.924	1.767	1.837	1.640	1.298
			P	0.012	0.110	0.035	0.145	0.015	0.001	0.296	0.328	0.104	0.018	0.003	0.000	0.014	0.010	0.010
	7B	AX-89703681	C/C	1.858	2.200	2.100	1.698	1.674	2.005	2.180	1.718	1.466	1.228	1.795	1.728	1.780	1.541	1.229
			T/T	1.996	2.305	2.319	1.735	1.847	2.191	2.326	1.911	1.564	1.347	2.019	1.879	1.907	1.707	1.373
			P	0.003	0.076	0.003	0.261	0.029	0.000	0.014	0.001	0.027	0.004	0.000	0.008	0.007	0.000	0.003
	7B	AX-109306202	C/C	1.860	2.224	2.129	1.636	1.676	2.044	2.205	1.773	1.520	1.231	1.883	1.743	1.756	1.614	1.224
			G/G	2.014	2.309	2.334	1.761	1.876	2.202	2.335	1.928	1.555	1.365	2.026	1.888	1.924	1.703	1.390
			P	0.000	0.073	0.000	0.004	0.003	0.000	0.007	0.000	0.194	0.000	0.000	0.002	0.000	0.004	0.000
	7D	AX-110913995	C/C	1.842	2.129	2.122	1.585	1.627	1.998	2.151	1.726	1.430	1.186	1.841	1.660	1.751	1.585	1.193
			T/T	2.073	2.402	2.421	1.839	1.975	2.285	2.417	1.999	1.632	1.438	2.090	2.006	1.984	1.749	1.460
			P	0.000	0.000	0.000	0.000	0.000	0.000	0.000	0.000	0.000	0.000	0.000	0.000	0.000	0.000	0.000
	7D	AX-110446329	C/C	1.991	2.382	2.324	1.768	1.858	2.228	2.356	1.945	1.603	1.367	2.019	1.939	1.929	1.714	1.390
			T/T	1.970	2.224	2.270	1.695	1.799	2.128	2.259	1.845	1.506	1.304	1.970	1.803	1.861	1.657	1.319
			P	0.284	0.001	0.175	0.042	0.188	0.002	0.024	0.011	0.004	0.028	0.106	0.001	0.036	0.033	0.033
	7D	AX-110967909	C/C	1.814	2.117	2.090	1.577	1.657	1.974	2.144	1.696	1.425	1.188	1.810	1.645	1.730	1.572	1.189
			T/T	2.070	2.398	2.406	1.819	1.936	2.275	2.398	1.995	1.632	1.416	2.091	1.989	1.981	1.754	1.453
			P	0.000	0.000	0.000	0.000	0.000	0.000	0.000	0.000	0.000	0.000	0.000	0.000	0.000	0.000	0.000
PH	1B	AX-111531574	G/G	98.367	104.006	94.253	93.722	108.596	95.991	102.852	86.582	95.306	99.656	90.767	105.613	86.546	97.814	103.391

			T/T	104.920	108.562	100.304	98.544	114.920	101.334	105.850	91.798	100.235	102.766	95.254	107.898	91.397	105.806	108.090
			P	0.003	0.015	0.005	0.007	0.002	0.005	0.047	0.007	0.007	0.069	0.003	0.128	0.005	0.000	0.021
	2D	AX-111132985	A/A	101.417	106.187	96.824	95.229	112.007	98.665	104.341	88.899	97.726	100.466	93.145	106.247	88.573	101.507	105.400
			C/C	107.012	109.324	102.523	103.673	115.692	102.185	105.824	94.087	100.729	104.905	95.644	108.709	94.057	109.979	109.836
			P	0.018	0.096	0.017	0.000	0.074	0.069	0.235	0.016	0.095	0.031	0.093	0.142	0.005	0.001	0.047
	4A	AX-109272207	C/C	108.347	112.113	103.820	101.181	118.615	104.354	108.793	94.826	102.790	106.463	98.139	110.556	94.663	108.737	111.396
			T/T	99.332	103.700	94.681	94.321	109.276	96.325	102.181	87.193	95.827	98.563	91.073	104.808	86.626	99.919	103.066
			P	0.000	0.000	0.000	0.000	0.000	0.000	0.000	0.000	0.000	0.000	0.000	0.001	0.000	0.000	0.000
	4A	AX-108745433	A/A	106.408	109.559	101.937	101.003	115.096	101.952	106.737	93.644	100.449	103.202	95.980	109.100	93.153	107.249	109.329
			G/G	99.745	104.746	94.874	93.333	111.446	97.590	103.282	86.687	96.682	99.929	92.212	104.726	86.651	99.703	103.760
			P	0.001	0.005	0.000	0.000	0.034	0.009	0.014	0.000	0.017	0.036	0.005	0.006	0.000	0.000	0.003
	4A	AX-111600193	A/A	99.689	104.643	94.939	94.577	110.517	96.863	102.642	87.507	96.346	99.293	91.603	105.145	87.200	100.113	103.704
			G/G	114.287	115.059	109.906	105.549	121.679	109.840	113.073	99.960	107.120	110.239	102.080	113.214	99.693	115.143	116.174
			P	0.000	0.000	0.000	0.000	0.000	0.000	0.000	0.000	0.000	0.000	0.000	0.001	0.000	0.000	0.000
	5B	AX-109860828	A/A	98.468	104.013	94.266	93.196	110.022	96.344	102.554	86.825	95.521	98.977	91.169	104.510	86.676	98.603	103.186
			G/G	115.439	115.745	109.868	108.043	120.575	109.152	111.213	99.475	107.260	109.537	101.699	114.167	99.075	117.460	116.507
			P	0.000	0.000	0.000	0.000	0.000	0.000	0.000	0.000	0.000	0.000	0.000	0.000	0.000	0.000	0.000
	5D	AX-95152512	C/C	101.534	105.867	97.058	95.624	111.747	98.458	103.909	88.765	97.633	100.312	93.109	105.978	88.693	101.976	105.249
			G/G	112.310	115.425	105.806	106.485	122.613	106.684	111.345	99.182	104.585	110.340	97.907	113.207	98.450	113.342	113.588
			P	0.002	0.002	0.009	0.000	0.001	0.006	0.004	0.001	0.013	0.001	0.033	0.010	0.001	0.001	0.010
	7B	AX-111568844	C/C	105.668	108.699	100.783	100.539	115.142	101.422	105.386	91.898	100.201	103.224	95.160	107.659	92.159	106.765	108.263
			T/T	98.547	104.313	94.281	91.915	109.838	96.702	103.379	87.089	95.927	98.911	91.684	105.406	86.415	98.234	103.390
			P	0.000	0.009	0.001	0.000	0.004	0.005	0.103	0.005	0.008	0.010	0.009	0.103	0.000	0.000	0.009
	7D	AX-109526545	A/A	101.604	106.061	97.102	96.359	111.936	98.623	103.981	89.206	97.780	100.638	93.049	106.395	88.746	102.272	105.264
			C/C	115.270	116.252	110.667	103.292	123.148	109.740	112.837	99.059	106.548	110.590	101.712	112.433	100.711	114.423	118.570
			P	0.000	0.002	0.000	0.017	0.001	0.001	0.001	0.003	0.004	0.002	0.001	0.035	0.000	0.001	0.000
SNPP	3B	AX-110142073	C/C	13.060	10.893	9.505	14.821	—	5.821	9.891	7.221	6.481	—	7.042	4.969	11.075	6.873	—
			G/G	13.599	11.365	10.753	16.586	—	6.222	10.128	7.492	6.515	—	7.450	5.128	12.043	7.382	—
			P	0.031	0.027	0.000	0.000	—	0.001	0.092	0.103	0.379	—	0.005	0.057	0.003	0.001	—

4A	AX-111600193	A/A	13.047	10.931	9.853	15.279	—	5.941	9.903	7.186	6.459	—	7.248	5.013	11.281	7.004	—
		G/G	14.253	11.748	10.755	16.888	—	6.325	10.488	7.977	6.656	—	6.994	5.140	12.514	7.431	—
		P	0.002	0.006	0.020	0.011	—	0.015	0.011	0.003	0.091	—	0.126	0.186	0.005	0.024	—
5A	AX-110033504	C/C	12.671	10.623	9.221	14.568	—	5.762	9.558	6.773	6.459	—	7.096	4.907	10.688	6.629	—
		T/T	13.865	11.591	10.861	16.631	—	6.263	10.461	7.895	6.537	—	7.343	5.186	12.408	7.556	—
		P	0.000	0.000	0.000	0.000	—	0.000	0.000	0.000	0.228	—	0.052	0.002	0.000	0.000	—
5B	AX-110586945	C/C	12.807	10.492	9.467	14.617	—	5.788	9.716	6.991	6.392	—	7.034	4.890	10.843	6.927	—
		T/T	13.637	11.524	10.552	16.382	—	6.198	10.226	7.582	6.584	—	7.350	5.170	12.127	7.259	—
		P	0.003	0.000	0.000	0.000	—	0.001	0.003	0.003	0.040	—	0.023	0.004	0.000	0.021	—
6B	AX-109084084	A/A	14.149	11.652	11.203	17.270	—	6.423	10.678	7.850	6.423	—	7.325	5.314	13.061	7.916	—
		G/G	13.029	11.032	9.839	15.331	—	5.917	9.851	7.231	6.513	—	7.187	4.989	11.215	6.935	—
		P	0.001	0.015	0.000	0.001	—	0.000	0.000	0.007	0.242	—	0.229	0.003	0.000	0.000	—
7B	AX-89703681	C/C	14.077	11.755	11.365	18.152	—	6.581	10.659	7.800	6.726	—	7.813	5.299	13.350	7.507	—
		T/T	13.179	11.026	9.909	15.263	—	5.927	9.913	7.268	6.443	—	7.111	5.010	11.252	7.055	—
		P	0.015	0.016	0.001	0.000	—	0.000	0.002	0.036	0.029	—	0.001	0.020	0.000	0.022	—
7B	AX-109306202	C/C	14.194	12.009	11.015	16.949	—	6.477	10.491	8.026	6.768	—	7.442	5.212	12.746	7.474	—
		G/G	12.979	10.819	9.822	15.333	—	5.868	9.884	7.106	6.404	—	7.134	4.988	11.198	6.997	—
		P	0.000	0.000	0.000	0.002	—	0.000	0.001	0.000	0.001	—	0.037	0.023	0.000	0.003	—
7D	AX-110913995	C/C	14.411	11.957	11.203	17.488	—	6.432	10.736	7.991	6.585	—	7.383	5.397	12.968	7.940	—
		T/T	12.501	10.469	9.211	14.314	—	5.741	9.487	6.825	6.439	—	7.087	4.804	10.550	6.544	—
		P	0.000	0.000	0.000	0.000	—	0.000	0.000	0.000	0.089	—	0.029	0.000	0.000	0.000	—
7D	AX-110967909	C/C	14.484	11.946	11.327	17.661	—	6.464	10.796	8.052	6.607	—	7.491	5.392	13.237	7.963	—
		T/T	12.581	10.603	9.319	14.506	—	5.765	9.555	6.875	6.466	—	7.059	4.844	10.544	6.618	—
		P	0.000	0.000	0.000	0.000	—	0.000	0.000	0.000	0.102	—	0.003	0.000	0.000	0.000	—

TKW, 1000-kernel weight; KNS, kernel number per spike; TSNS, total spikelet number per spike; GWS, grain weight per spike; PH, plant height; SNPP, spike number per plant. ‘–’, indicated that data were missing

TABLE S8 Cultivars with more than 20 favorable alleles and their traits.

Accession No.	Name	No of superior alleles	FSNS	TKW	KNS	GWS	SL	TSNS	SNPP	PH
ZM009469	Zhengzhou683	30	19.06	44.34	50.66	2.29	8.53	20.19	7.52	103.13
ZM014971	Suzhou7829	29	17.68	42.14	49.32	2.11	9.17	19.08	7.63	96.45
ZM009825	Longchun7	28	18.65	42.36	56.40	2.42	10.40	20.09	7.25	111.28
ZM014848	Ningmai6	28	18.43	37.66	52.37	1.98	8.07	20.15	8.62	81.87
ZM016966	Yunmai35	28	19.81	40.98	49.92	2.11	9.44	20.75	8.56	119.95
ZM015139	Zhen7630	28	16.87	42.27	46.08	1.97	7.59	18.07	7.73	85.78
ZM010516	Wanya2	27	17.84	38.97	50.35	1.97	9.46	19.02	6.93	89.27
ZM016677	Chuan533	26	18.56	40.31	48.20	1.97	7.46	19.43	8.73	96.02
ZM016678	Chuan578	26	17.51	44.40	47.06	2.11	9.00	18.72	8.98	91.31
ZM016373	E31846	26	17.64	39.98	52.69	2.13	9.10	17.62	6.68	89.91
ZM009927	Linmai7	26	17.39	43.37	47.49	2.10	10.27	19.32	7.54	107.85
ZM026716	Yangmai15	26	18.25	45.84	47.92	2.22	7.80	19.37	8.10	70.94
MY003143	Wilhelmina	25	21.83	28.75	44.63	1.25	9.25	23.60	8.96	118.43
ZM016876	Bimai6	25	20.28	35.23	50.36	1.82	9.58	21.57	9.73	83.74
ZM018320	Gan(39)-2	25	20.69	42.76	59.99	2.62	10.71	21.48	6.31	107.19
ZM009708	Kehan2	25	19.13	34.81	41.64	1.54	12.05	22.27	10.98	132.68
ZM009924	Linmai4	25	17.52	41.43	49.10	2.10	10.62	18.97	8.21	108.73
ZM009909	Linnong12	25	19.69	39.55	59.30	2.41	9.88	20.91	7.22	100.82
ZM022994	Nanzhao76144-0-6-1	25	18.39	38.80	50.78	1.98	8.26	19.26	7.48	84.34
ZM010194	Ningmai3	25	19.16	35.79	57.10	2.09	9.00	19.95	8.22	89.39
ZM017206	Shannong21-24	25	19.50	32.89	57.29	1.94	9.26	21.37	8.30	120.65
ZM010478	Xichang662	25	18.48	35.23	59.08	2.14	11.02	19.26	7.60	109.18
ZM026713	Yangmai12	25	18.50	41.75	48.83	2.05	9.37	20.06	9.24	84.80
ZM026715	Yangmai14	25	17.31	45.01	47.01	2.13	9.65	18.46	7.87	81.37
ZM014902	Yangmai4	25	18.13	42.11	50.51	2.18	8.28	19.38	7.31	96.00
ZM009472	Zhengyin1	25	18.11	37.04	52.95	2.00	8.83	19.11	8.30	89.21
ZM009465	Zhengzhou17	25	19.21	41.98	54.52	2.35	9.63	20.64	7.52	115.62
ZM009580	Zhuyeqing(white)	25	17.91	37.60	48.08	1.87	8.77	19.66	9.15	116.71
ZM023194	Chuan80-466	24	19.86	42.20	50.78	2.20	9.04	21.02	8.56	86.07
ZM016503	Emai9	24	18.40	43.09	53.25	2.34	9.46	19.12	7.80	106.80
ZM017265	Gan81(39)-2-7	24	20.54	42.31	60.00	2.64	11.06	21.51	6.54	110.95
ZM010319	Huamai7	24	17.85	40.51	43.40	1.81	9.75	19.44	8.93	109.38
ZM022203	Jurong03	24	17.73	38.67	46.14	1.80	8.39	18.68	8.41	81.04
ZM009502	Kaifeng10	24	19.75	39.12	49.44	1.98	7.35	20.84	8.65	105.06
ZM009719	Kezhen	24	21.41	28.58	55.94	1.64	14.19	24.75	8.51	132.46
ZM009907	Linmai5	24	-	40.10	64.90	2.60	12.60	21.20	13.70	126.90
ZM009908	Linnong11	24	20.01	38.65	61.55	2.41	9.17	21.32	6.54	98.60
ZM009910	Linnong13	24	20.21	39.48	56.24	2.25	9.46	21.27	7.86	107.79
ZM009905	Linnong1	24	18.69	40.24	52.84	2.17	11.17	20.15	7.18	110.92
ZM009906	Linnong2	24	18.92	37.40	52.82	2.02	10.26	20.46	8.42	107.61
ZM009494	Mengxian2	24	18.88	38.45	54.29	2.12	8.63	20.08	9.41	70.97

ZM010392	Ningmai1	24	18.77	36.49	48.45	1.83	10.03	20.40	8.48	113.93
ZM022235	Yangmai158	24	17.56	43.87	51.22	2.27	9.09	18.61	7.82	86.80
ZM026717	Yangmai16	24	19.22	43.68	56.76	2.50	9.43	20.27	7.77	82.48
ZM026718	Yangmai17	24	18.26	37.74	52.02	1.96	9.45	19.56	9.65	86.32
ZM010480	Youyimai	24	19.36	33.84	50.54	1.73	9.05	20.79	10.01	79.90
ZM010547	Yunmai27	24	19.85	38.52	50.29	1.99	9.93	21.47	8.87	103.37
MY000161	Abbondanza	23	18.41	41.41	50.26	2.08	10.87	19.98	8.78	115.01
ZM015488	Ai73	23	19.70	29.81	60.38	1.83	9.65	21.24	8.35	94.05
ZM010197	Aiganzao	23	17.54	34.15	49.09	1.70	6.54	18.68	9.15	99.18
ZM010450	Fan6	23	20.64	34.43	59.63	2.10	7.41	20.64	8.54	85.62
ZM022851	Guangyang821	23	18.51	42.38	52.55	2.27	9.09	19.58	7.73	82.06
ZM008937	Jinghong9	23	17.99	32.15	48.01	1.59	10.02	19.62	9.59	109.11
ZM009926	Linmai6	23	19.81	37.31	60.81	2.31	10.06	20.77	6.86	110.28
ZM009911	Linnong14	23	20.99	38.81	63.36	2.54	9.75	21.26	6.96	105.42
ZM022052	Long90-05634	23	20.89	28.29	51.21	1.45	12.65	22.66	9.41	117.03
ZM010464	Mianyang4	23	19.57	34.02	54.13	1.86	9.86	20.86	8.11	110.84
ZM010589	Rikaze8	23	17.86	36.43	42.46	1.59	9.53	19.66	9.17	117.05
ZM017165	Shan6815-0-2-3	23	15.98	38.22	44.63	1.73	10.00	17.19	10.80	87.93
ZM009492	Shicha15	23	19.22	42.31	48.83	2.11	10.33	20.84	7.72	85.51
ZM014926	Suyang8-1-2	23	19.28	38.13	56.31	2.17	9.23	20.72	8.44	91.45
ZM009189	Taiyuan567	23	17.97	39.93	44.94	1.83	7.46	19.86	10.06	82.42
ZM017079	Xiaoyan6	23	17.42	39.29	40.61	1.62	7.56	19.60	11.10	95.61
ZM016194	Xuchang26	23	18.96	46.09	53.13	2.48	10.37	20.37	7.35	114.66
ZM026711	Yangmai10	23	19.22	43.51	57.88	2.56	9.70	20.27	7.73	81.95
ZM026712	Yangmai11	23	17.87	45.43	48.55	2.31	8.65	19.32	8.15	86.06
ZM026714	Yangmai13	23	19.25	40.00	58.64	2.36	10.47	20.04	7.37	77.55
ZM025137	Yangmai9	23	19.15	40.93	50.78	2.09	8.94	20.11	9.45	74.95
ZM010545	Yunmai25	23	20.30	38.77	53.45	2.12	8.50	21.44	7.57	104.02
	Zhengyin4	23	19.14	34.20	48.40	1.69	8.01	20.61	10.08	78.66
ZM009470	Zhengzhou722	23	18.56	40.80	51.92	2.15	8.05	19.92	8.40	91.16
ZM009471	Zhengzhou742	23	18.11	42.55	54.24	2.31	9.70	19.81	7.64	86.59
ZM009977	Zhongliang11	23	19.62	34.73	49.96	1.78	10.09	21.04	11.44	124.90
ZM009581	Zhuyeqing (red)	23	17.40	37.23	46.25	1.75	8.36	19.26	9.05	113.40
MY001212	Heine Hvede	22	20.92	34.27	46.64	1.66	8.97	23.48	9.38	108.18
ZM010370	Baimangmai	22	17.71	43.50	45.41	2.02	8.37	19.14	8.58	111.80
ZM016378	E34963	22	17.92	35.10	52.36	1.87	10.18	19.55	9.07	95.64
ZM010314	Emai6	22	18.53	40.28	51.81	2.16	8.84	19.72	9.35	110.33
ZM015557	Fufan16	22	19.77	40.61	60.26	2.47	9.15	20.75	6.64	99.64
ZM010327	Jingzhou1	22	18.19	41.44	46.89	1.99	10.91	20.18	8.24	119.27
ZM010332	Jingzhou66	22	19.10	37.35	47.65	1.82	9.99	20.21	8.51	112.64
ZM009506	Kaizhong70-18	22	18.99	44.82	45.50	2.09	8.36	20.36	8.04	96.89
ZM014681	Kehan9	22	18.65	31.66	40.63	1.32	11.67	21.23	9.28	125.32
ZM010211	Nanda8	22	17.84	38.35	44.46	1.74	8.12	18.61	8.83	102.46
ZM009526	Neixiang173	22	17.60	39.09	46.93	1.86	7.44	18.98	8.67	89.94

ZM026690	Ningmai8	22	19.97	38.00	55.53	2.20	8.55	20.67	6.97	71.19
ZM024772	Shanyou225	22	17.92	38.87	44.93	1.75	8.00	19.18	8.76	77.62
ZM010490	Shuwan8	22	19.79	37.47	50.04	1.96	8.94	20.51	8.04	107.24
ZM010306	Wannian2	22	17.66	36.73	45.69	1.73	9.59	19.22	8.45	117.58
ZM010243	Wangmai15	22	19.07	36.43	47.65	1.80	10.21	20.13	9.14	125.39
ZM017209	Xiannong151	22	17.87	37.44	53.34	2.02	8.77	19.22	8.22	77.39
ZM009508	Xinzheng1	22	18.17	34.83	48.13	1.74	7.12	19.43	7.83	93.10
ZM016120	Yu30691-3-6 (white)	22	17.73	37.79	53.34	2.07	10.47	19.45	9.29	89.43
	Yuanfeng175	22	18.85	38.09	49.06	1.87	7.49	20.05	8.24	73.75
ZM015988	Zhengzhou741	22	18.47	38.79	54.60	2.12	8.76	19.81	8.18	78.71
ZM015989	Zhengzhou761	22	18.16	39.36	43.05	1.70	8.02	19.10	8.52	76.12
MY001072	Funo	21	19.48	34.98	54.33	1.93	8.84	21.04	9.31	99.20
ZM016703	Chuan78001	21	19.01	38.19	54.12	2.04	8.02	19.69	7.31	73.35
ZM016330	E1161	21	18.61	38.94	52.31	2.08	8.27	19.60	7.25	92.55
ZM024056	Fengqiang7	21	18.86	38.56	47.28	1.91	11.93	20.17	8.38	123.20
ZM009802	Ganmai7	21	18.32	39.55	52.43	2.12	10.27	19.60	7.38	117.34
ZM022170	Hei86-30	21	18.92	31.66	52.54	1.68	12.65	20.96	9.72	115.66
ZM015946	Huapei126	21	19.44	44.32	50.40	2.26	9.68	20.67	8.41	96.84
ZM015948	Huapei128	21	20.58	30.15	59.20	1.82	10.28	22.03	8.83	70.06
ZM015126	Jian37	21	20.19	37.42	58.57	2.21	9.58	20.77	7.29	97.58
ZM011384	Jinguangmai	21	19.34	38.18	46.10	1.76	11.64	21.20	9.54	113.85
ZM015036	Jinling1	21	17.76	35.22	48.69	1.74	8.04	19.02	10.21	79.17
ZM010374	Jinmai33	21	19.19	37.39	53.41	2.01	9.79	20.65	8.02	99.18
ZM022722	Lumai17	21	16.61	45.55	39.55	1.84	10.18	18.27	10.46	94.95
MY002004	Mexipak66	21	18.84	30.79	59.76	1.93	10.37	20.24	8.58	93.63
ZM017138	Shan5860-19-2-2	21	20.22	36.19	50.14	1.86	9.06	21.89	8.87	118.96
ZM017162	Shan6801-3-1-1	21	17.22	39.37	45.23	1.79	9.96	19.64	8.94	82.76
ZM017180	Shan7587-1/81	21	19.13	38.75	56.33	2.22	8.95	20.29	10.13	88.88
ZM009520	Suinong3	21	18.21	39.00	51.47	2.04	7.60	19.40	8.33	90.28
ZM009616	Xianfeng68	21	20.00	31.90	58.42	1.88	7.21	20.97	8.56	83.50
ZM016512	Xiangmai5	21	18.61	39.04	53.13	2.07	8.82	19.95	8.18	83.42
ZM010221	Xinfeng13	21	18.30	33.91	55.51	1.87	8.10	19.19	8.24	96.30
ZM009528	Xinyang12	21	18.72	39.87	51.33	2.10	10.26	19.77	8.65	111.02
ZM017950	Xinyang1	21	17.89	37.70	47.53	1.84	7.65	19.13	9.07	113.66
ZM015104	Xuzhou20	21	19.47	39.70	54.03	2.16	8.77	19.88	8.32	83.31
ZM025075	Yang92-90	21	18.80	39.30	50.86	2.03	8.03	20.53	6.98	88.95
	Zhengmai9023	21	16.46	46.87	42.74	2.00	9.84	18.83	8.27	77.37
ZM015995	Zhengzhou6811	21	18.81	43.05	53.26	2.38	9.37	20.31	9.37	119.53
ZM017939	Zhengzhou743	21	18.07	41.48	51.87	2.22	9.62	19.57	7.62	86.84

SL, spike length; TKW, 1000-kernel weight; KNS, kernel number per spike; TSNS, total spikelet number per spike; FSNS, fertile spikelet number per spike; GWS, grain weight per spike; PH, plant height; SNPP, spike number per plant. '–', indicated that data were missing
